# Towards energy-efficient joint relay selection and resource allocation for D2D communication using hybrid heuristic-based deep learning

**DOI:** 10.1038/s41598-025-08290-x

**Published:** 2025-07-12

**Authors:** C. H. Ramesh Babu, S. Nandakumar

**Affiliations:** School of Electronics Engineering, Vellore Institute of Technology, Vellore, India

**Keywords:** Adaptive residual gated recurrent unit, Device-to-device communication, Hybrid manta-ray foraging with chef based optimization, Joint relay selection and resource allocation, Engineering, Electrical and electronic engineering

## Abstract

Fifth generation (5G) networks are desired to offer improved data rates employed for enhancing innovations of device-to-device (D2D) communication, small base stations densification, and multi-tier heterogeneous networks. In relay-assisted D2D communication, relays are employed to minimize data rate degradation when D2D users are distant from one another. However, resource sharing between relay-based and cellular D2D connections often results in mutual interferences, reducing the system sum rate. Moreover, traditional relay nodes consume their own energy to support D2D communication without gaining any benefit, affecting network sustainability. To address these challenges, this work proposes an efficient relay selection and resource allocation using the novel hybrid manta ray foraging with chef-based optimization (HMRFCO). The relay selection process considers parameters like spectral efficiency, energy efficiency, throughput, delay, and network capacity to attain effectual performance. Then, the data provided as the input to the adaptive residual gated recurrent unit (AResGRU) model for the automatic prediction of an optimal number of relays and allocation of resources. Here, the AResGRU technique’s parameters are optimized by the same HMRFCO for improving the prediction task. Finally, the designed AResGRU model offered the predicted outcome.

## Introduction

Wireless communication networks should start the evolution to maintain the emerging requirement of large data rates because of growing mobile communication applications. With the enhancement of wireless communication, the spectrum resource requirement is developing^1^. With the development of 5G wireless devices, the wide-scale intelligent system’s access relatively results in the spectrum resource shortage. With artificial intelligence (AI), virtual reality, the medical sector, and voice detection applications in the sector of mobile communication, the computation operations result in much demand on the smart system’s data access and computation capacity^2^. But, restricted by the finite amount of spectrum resources, sending directly the offloading approach to the edge server leads to an enhancement in the smart system’s transmission energy in terms of transmission distance, bandwidth, and other attributes such as computation offloading cannot obtain higher energy efficacy^3^. The wide scale of transmission information cannot be ended because of the lower transmission rate of the data and latency factor, though mobile edge computing (MEC) offers high potential computational ability. Hence, edge caching is recommended to prevent unwanted redundant tasks and data transmission^4^. But the mobile application’s input data is continuously upgraded and the continuous information caching enhances the communication overhead hence the high energy utilization problem cannot be rectified the sustainability. Enhancing the transmission ability is a significant solution^5^. Thus, the D2D communication innovation is recommended to mitigate the issue properly. To enable the distance smart systems to employ the MEC server’s computational resource, a powerful relay must be supported for achieving the computing data’s transmission^6^.

In recent days, the communication in D2D has become highly popular because of its significant merits, numerous of that have been examined in research works^7^. The communication in D2D defines the direct communication technology among two cellular systems without subjecting via the base station. This assists to tune some cellular traffic to the D2D frameworks, improving the capacity of the network, enhancing the efficacy of spectrum, decreasing the latency, and scaling coverage^8^. These merits make D2D communication highly applicable for the applications of social networking such as media sharing and wireless video streaming. Effective resource allocation offers a significant part in understanding the merits of D2D communications in improving spectral efficacy^9^. Most importantly, it is suitable to enable efficient D2D transmissions while ensuring the Quality of Service (QoS) of conventional cellular communications. The D2D communication’s direct model cannot be reliable because of the channel interferences and fading from cellular connections. Hence, it is significant to utilize the D2D relaying transmissions or the D2D connections in undesired transmission situations to improve the rates and reliability of the communication^10^. In addition, the D2D transmission’s resource allocation has attained high focus in the research sector^11^.

But, the D2D communications gives several limitations including energy consumption and spectrum sharing. Thankfully, the research sector is addressing these limitations^12^. With the relationship of wide-scale wireless systems, the consumption of global energy is enhanced further, and various experiments have been conducted to enhance energy efficacy and minimize consumption^13^. The resource allocation and selection of mode for the D2D connections is implemented; however, it does not focus on the mode of relay^14^. There are some other conventional techniques concentrating on the resource allocation for communication of D2D utilizing relays. However, the conventional techniques force the D2D connections to transmit via relays that cannot lead to better network functionality since the straight mode is a good option for effective D2D connections^15^. Nowadays, machine learning strategies have been employed to choose the relays with good functionality rates. According to the relay sources, nodes, and secrecy rates of the destination, machine learning strategies are largely employed^16^. But, the strategies of machine learning employ the evaluation factors such as bit-error probability, outage likelihood, and capacity to estimate the functionality are not focussed here^17^. As an outcome, energy efficacy is one of the numerous complexities in the future generation communication models. D2D communication enhances spectrum efficiency and reduces the latency in 5G networks. But it faces key challenges such as unreliable direct links and severe interference due to spectrum sharing. So, the relay assisted communication and resource allocation are useful to address this issue. However, the joint optimization of relay selection and resource allocation is a complex, multi-objective problem. It requires adaptive and scalable solutions. This paper proposes a hybrid optimization algorithm to improves the overall system performance in relay assisted D2D communication.

The implemented joint relay selection and resource allocation task includes the following contributions.

- To construct the joint relay selection and resource allocation task by utilizing hybrid heuristic-based deep learning that helps the D2D communication systems to perform error-free and rapid data transmissions.
- To design an AResGRU technique for performing the automatic prediction of an optimal number of relays and resource allocation. Here, the HMRFCO is supported for tuning the AResGRU technique parameters.
- To select the relays and resources optimally by utilizing the recommended HMRFCO approach that maximizes the energy and spectral efficiency, and minimizes the delay. Moreover, it supports to improve the network capacity and throughput.
- To generate a new HMRFCO approach by hybridizing the conventional CBOA and MRFO approaches that help to optimally choose the relays, and resources in the D2D communication system.
- To examine the robustness and efficacy of the suggested approach by employing various performance metrics, traditional techniques, and algorithms.

The rest of the presented work is organized as given. Module II offers the literature works on the presented joint relay selection and resource allocation task in a D2D communication system. Module III elucidates the HMRFCO approach for parameter tuning in the D2D model. Module IV offers the system model of D2D communication and the description of sum rate and energy efficiency. Module V forecasting the resource and relay in the D2D model employing AResGRU and multi-objective function.

## Existing works

### Related works

Chen et al.^18^ have analyzed the issue of energy-efficient resource allocation in D2D communication. This work concentrated on improving the overall energy efficiency in entire D2D pairs while ensuring the secrecy rates and QoS demands. Dinkelbach’s algorithm was employed to convert the real fractional programming issue into a subtractive form. The simulation outcomes displayed that the model displayed superior functionality contrasted with other approaches.

Lee and Schober^19^ have suggested a deep learning mechanism for resource allocation optimization in D2D communication. Instead of resolving the resource allocation issue for each channel, the deep learning model was suggested, where the optimal resource allocation mechanism was suggested by employing deep neural network (DNN) techniques. The simulation outcomes ensured that the near-optimal performance was attained with minimal computational period.

Zhang et al.^20^ have suggested a system to decrease the overall transmission power utilization of the D2D connections. Experts derived the optimization issues and further implemented a distributed mechanism on the basis of game theory to rectify them. The simulation outcomes illustrated that the author’s task could highly minimize the transmission energy utilization contrasted with conventional tasks.

Salim et al.^21^ have recommended a low-complexity approach that estimated the reuse partners and offered two distinct mechanisms for selecting the optimal relay. The experiments displayed the suggested model’s behaviour under diverse attributes and its promising functionality when contrasted to one of the modern techniques concerning the energy efficacy of the relay and the sum rate of the links.

Wang et al.^22^ have implemented a multi-relay technique and further evaluated the cooperation behaviours. Next, a simple mechanism was recommended to rectify the issue of relay selection, and further effective resource-sharing issues were derived. The fairness and the system efficacy were ensured by several simulation outcomes.

Gu et al.^23^ have discussed the real-aided D2D communication from two views. At first, the relay selection issue was analyzed. A two phase’s relay selection as well as resource allocation mechanism was implemented. In the second phase, this work discovered that the two stages approach created a throughput unbalance issue. Hence, a throughput balance mechanism was implemented. The simulations displayed that the two-stage mechanism could improve the throughput of the relay-aided D2D communication.

Tian et al.^24^ have validated the joint relay selection as well as the resource allocation mechanism for the relay-based D2D communication models. The goal of this work was to improve the system’s overall transmission rate while ensuring the QoS demands. A social-aware relay selection method was implemented with minimum computational burdens for achieving the proper relay nodes to the D2D connections. The numerical solutions demonstrated that this mechanism was capable of enhancing the functionality of the system contrasted to other approaches.

Lyu et al.^25^ have concentrated on the resource allocation issue during the power transfer operation and the issue of resource allocation during the operation of data transmission. A credit approach was suggested for the resource allocation issue. Moreover, a Stackelberg differential game-aided approach was suggested for the issue of resource allocation. Through extensive validations, the efficacy of the model was validated.

Gui et al.^26^ have recommended an energy-efficient resource allocation mechanism for estimating the power allocation and channel selection by suggesting the non-cooperative game theory and a relay-oriented D2D communication system. This work mitigated the inter-cell interference and also reduced the interfering signal processing load. The energy efficiency was enhanced because of the transmission power minimization. The developed model achieved higher performance.

Ali et al.^27^ have derived the issue of energy efficiency maximization concerning the cell selection and resource allocation of the HetNet’s. The goal was to increase the throughput. This work recommended an Outer Approximation Algorithm (OAA) to rectify the issue of converted concave optimization. The model was estimated by extensive simulation tasks. The high functionality was achieved with the presence of distinct network parameters.

Feng et al.^28^ have discovered a new mechanism that integrated with the energy harvesting technology and D2D communication. The author’s objectives were to examine the enhancement of energy efficacy of the model by selecting relays and jointly allocating time. Hence, by employing the fractional programming theory, experts recommended the iterative optimization mechanism to rectify the convex optimization issue for attaining a better outcome. The outcomes displayed that the designed approach was highly enhanced contrasted with the baseline techniques.

Salim et al.^29^ have examined the Energy Harvesting (EH)-assisted two-way relaying (TWR) D2D communication distributing the conventional cellular network’s spectrum. This work recommended an energy efficiency (EE) trade-off EH-aided model (REET-EH). This model could do the resource allocation, relay selection, and power allocation optimally. The numerical outcomes displayed that the model enhanced the system functionality with other approaches.

Pasha et al.^30^ have presented a deep learning-based hybrid resource allocation framework that combines a metaheuristic hybrid particle swarm technique with a modified long short-term memory (LSTM) model. This strategy seeks to enhance the system capacity and optimize power control while taking quality of service limitations into accounts.

Liu et al.^31^ have suggested a joint resource allocation and the selection of a drone’s relay framework concentrating to enhance the sum rate of the D2D device while confirming the QoS demands for the D2D and cellular candidates. This work utilized a new approach for minimizing the computational complexity and offered an in-depth evaluation of the recommended work. The experimental solutions portrayed that the suggested framework enhanced the system functionality.

Li and Chen^32^ have presented a new resource allocation scheme for the D2D networks on the basis of enhanced Monte Carlo Tree Search (MCTS). In this work, optimal classification theory was employed to rectify the transmit power of the user. The simulation experiments explained that the suggested work offered better solutions than the conventional techniques.

Chauhan and Gupta^33^ have analysed the conventional approaches for D2D transmission pairs. By analysing these approaches, the authors implemented a new technique for the D2D networks. The recommended work enhanced the performance rates in any situation and then the authors performed the resource allocation. The experiments were carried out and realized that the model offered higher throughputs.

Li et al.^34^ have recommended a resource allocation and mode selection mechanism for D2D communication networks concentrating to attain a balance of user satisfaction and throughput. In order to store the offline channel state information, a technique was implemented on the basis of geographic location. The differential evolution approach was utilized in this work and estimated the user satisfaction. The outcomes explained that the recommended framework attained satisfactory outcomes.

Hussain et al.^35^ have implemented a new algorithm for rectifying the issues in the resource allocation in D2D networks. The research experiments displayed that the recommended approach outperformed the conventional techniques in estimating the specific power levels while facing the corresponding constraints, highly enhancing the capacity of the system and minimizing the interference.

Gopal^36^ has offered a sequential mechanism to reutilize the cellular-category resources and reduce the delay for the D2D users. This work presented a new optimization approach to enhance the throughput of the network. The presented work concentrated to improve the entire spectrum efficacy and throughput of the network. The simulation experiments were carried out and outcomes illustrated that the recommended work attained higher efficiency.

Gopal and Velmurugan^37^ have implemented a hybrid mechanism for joint uplink and downlink to improve the throughput and allocate the resources. The optimization issue was derived as a mixed-integer non-linear issue that was normally NP-hard. The recommended technique could enhance the spectrum efficiency and throughput. The numerical analysis implied that the recommended task effectively outperformed the traditional techniques.

An et al.^38^ have modelled a resource allocation for the D2D device as a constrained optimization issue. Further, the suggested technique’s variable-coupling relationship was evaluated and the mathematical proof was provided. The effectiveness of the recommended task was validated and the experiments ensured that the suggested system provided the accurate solutions for the resource allocation tasks.

Jin et al.^39^ have explained about Unmanned Aerial vehicles (UAEs) for the next generation of wireless networks 5G/6G. Unfortunately, the low-power batteries that power the UAVs that are now in use severely restrict their operating life, leading to different degrees of communication disruptions and increased costs. As a result, increasing UAV communication’s energy efficiency (EE) has emerged as a critical issue that requires immediate attention. This article provides a thorough analysis of techniques to increase UAV energy efficiency (EE), including resource allocation and management, energy-saving communication protocol design, and UAV trajectory planning and deployment.

Gouda et al.^40^ suggested a subchannel assignment method to enhance the dynamic UAV-assisted cellular networks performance. By utilizing the dynamic hypergraph colouring, this method provides the best subchannel assignment while considering the social ties and interference into account. The simulations results showed that the suggested method has provided the better system throughput, energy efficiency and interference efficiency.

Austine and Pramila^41^ have implemented a hybrid approach combining flow direction algorithm (FDA) and chameleon swarm algorithm (CSA) to optimize the relay selection and resource allocation in D2D communication. The simulation results shows that the suggested algorithm enhances the sum rate and minimizes the mutual interference.

Austine, et al.^42^ have explained a genetic algorithm-adaptive bat optimization (GA-ABO) model for efficient resource allocation in cellular networks with D2D communication. The methods aims to enhance the throughput and reduce the interference.

Austine et al.^43^, in order to maximize spectrum allocation, power control, and link matching in cooperative D2D communications, this study investigates a hybrid strategy that combines centralized and distributed systems with deep reinforcement learning. The technique seeks to improve network performance and energy efficiency.

### Research gaps and challenges

Joint optimization of relay selection and resource allocation can result in improved overall effectiveness in D2D communication. By synchronizing the allocation of assets and choosing the best relays, the network may make better use of the resources at hand, leading to improved efficiency and faster data rates. However, it adds intricacy to the whole thing, necessitating more advanced methods and processing resources. This variety can make systems more difficult to design and upkeep, which could lead to greater expenses and efficiency concerns, as well as increased communication and management overhead when exchanging data among equipment and the network as a whole. The features and changes of the existing joint optimization of relay selection and resource allocation models are given in Table 1. The section as shown below offers the research gaps.

- There is a need for more efficient algorithms that can handle the complexity of joint optimization in relay selection and resource allocation.
- Research is needed to develop energy-effective resource allocation techniques that consider the limited energy resources of D2D devices.
- There is a requirement to address the limitations encountered by dynamic network conditions, such as varying channel conditions and user mobility, in the joint optimization process.
- Research is needed to develop scalable solutions that can handle a large number of D2D users and optimize resource allocation and relay selection in such scenarios.
- More research is needed to incorporate different Quality of Service (QoS) demands of D2D candidates into the joint optimization process, considering factors like latency, reliability, and throughput.

**Table 1:** Features and challenges of the joint optimization of resource allocation and relay selection in D2D communication.

References	Methodology	Features	Challenges
Chen et al.^18^	Dinkelbach’s algorithm	It helps to discover the optimal solution and enhance the performance of the overall system It is robust to variations in the system parameters such as the number of systems, and interference levels	It increases the computational complexity and processing time Its scalability is limited and sensitive to initial conditions
Lee and Schober^19^	DNN	It can understand the difficult patterns and allocate the resources robustly It has high flexibility and handles large-scale systems effectively	It requires more computational resources for effective training It has limited interpretability and generalization capability
Zhang et al.^20^	TRSPC	Increases spectrum and energy conservation in D2D interactions Allows for adaptive transmission pace choice and energy regulation, which improves the allocation of resources and relaying choice	Needs correct channel status data and complex computations for rate choice and control of power
Salim et al.^21^	RPRS-EH	Enhances resource allocation and battery management simultaneously to increase system efficiency	Necessitate advanced strategies for optimization and actual time network data, which may increase computing costs
Wang et al.^22^	Multi-relay system model	Allows for more freedom in relay selection according to network circumstances and the needs of users Offers for collaborative interaction across several relays, improving range and dependability in D2D systems	A drawback is the greater difficulty in relay choice and cooperation that may result in higher signaling overhead
Gu et al.^23^	Throughput balance scheme	It increases each system’s throughput and minimizes the interference among the systems It enhances the QoS of each system and has high scalability	It is not optimal for all scenarios and demands additional hardware resources It is not robust to channel variations, which affects the accuracy
Tian et al.^24^	Social-aware relay selection algorithm	By choosing the optimal relays, this model improves the energy efficiency and throughput of the system It improves the relay selection process by focusing on the social relationships and the trust among the systems	It is not suitable for real time applications and is prone to social engineering threats It demands additional information that is hard to achieve
Lyu et al.^25^	Stackelberg differential game-based model	It can be utilized in distinct scenarios and provides high efficiency It improves resource allocation by concentrating on the strategic interactions among the systems	It is not robust to variations in the network topology It gives poor performance when processing large scale systems
Gui et al.^26^	Non-cooperative game theory	It helps to make decisions on the basis of its own self-interest thus improving the system’s performance It minimizes the system overhead complexities	It takes more computational time to perform the resource allocation It demands additional strategies to guarantee fairness
Ali et al.^27^	OAA	It reduces the computational burdens and is robust to noise It can converge faster to the optimal outcome contrasted to other algorithms	It fell into the local optimum trap and utilized more time to complete the iteration It has limited scalability
Feng et al.^28^	EHA-CRD	Cognitive abilities allow smart allocation of resources and relay choice in response to supply and network circumstances Uses methods of energy harvesting for powering D2D gadgets, decreasing reliance on outside power supplies	Climatic conditions may limit power collection, resulting in inconsistent power supply and communications problems
Salim et al.^29^	REET-EH	It enhances the energy efficiency and network lifetime It improves the QoS by guaranteeing that the systems have the proper energy to transmit the data	It is not applicable to systems with limited EH capabilities It struggles to provide better solutions with the system interferences

Thus, effective joint optimization of relay selection and resource allocation is provided in this work for communication in D2D, and Table 1 offers the advantages and limitations of conventional joint optimization of relay selection and resource allocation approaches in D2D communication.

## Hybrid manta-ray foraging with chief-based algorithm for parameter tuning used in D2D model

### Conventional algorithm: MRFO

Numerous real-world optimization issues are relatively becoming difficult. Meta-heuristic approaches for managing the complexity of optimization issues are highly becoming famous. One of the nature-motivated optimization mechanisms is MRFO^14^, which offers a different optimization strategy for rectifying real-world optimization limitations. The intelligence of manta rays is considered in the existing MRFO. The manta rays have special foraging properties to resolve diverse optimization limitations. The MRFO’s functionalities are given as mathematically here.

The foraging properties including “chain foraging, cyclone foraging, and somersault foraging” are considered in this strategy.

*“Chain foraging”* The manta rays are capable to monitor the plankton’s regions and going towards them. The plankton’s concentration decides the particular region’s effectiveness. Automatically, the MRFO takes the best outcome obtained so far. This phase is expressed in Eqs. (1) and (2).

1 $$l_{m}^{p} \left( {a + 1} \right) = \left\{ {\begin{array}{*{20}l} {l_{m}^{p} \left( a \right) + j\cdot \left( {l_{bt}^{p} \left( a \right) - l_{m}^{p} \left( a \right)} \right) + \alpha \cdot \left( {l_{bt}^{p} \left( a \right) - l_{m}^{p} \left( a \right)} \right)} &\quad {m = 1} \\ {l_{m}^{p} \left( a \right) + j\cdot \left( {l_{m - 1}^{p} \left( a \right) - l_{m}^{p} \left( a \right)} \right) + \alpha \cdot \left( {l_{bt}^{p} \left( a \right) - l_{m}^{p} \left( a \right)} \right)} & \quad {m = 2,\ldots ,X} \\ \end{array} } \right.$$

2 $$\alpha = 2\cdot j\cdot \sqrt {\left| {\log \left( j \right)} \right|}$$

In this, the weight coefficient is defined by α and the *m*th manta ray’s region at a time *a* is specified by $l_{m}^{p} \left( a \right)$ in *p*th dimension. The highly concentrated plankton is taken as $l_{bt}^{p} \left( a \right)$ and the random vector is taken as *j* in the limit of 0 and 1.

*“Cyclone foraging”* If the plankton is found underwater, the manta rays swim toward it in a spiral form. The groups of manta rays are designing spirals to conduct the foraging. The cyclone foraging is expressed in Eq. (3) for the 2D space.

3 $$\left\{ {\begin{array}{*{20}l} {L_{m} \left( {a + 1} \right) = L_{bt} + j\cdot \left( {L_{m - 1} \left( a \right) - L_{m} \left( a \right)} \right) + e^{xc} \cdot \cos \left( {2\pi c} \right)\cdot \left( {L_{bt} - L_{m} \left( a \right)} \right)} \\ {K_{m} \left( {a + 1} \right) = K_{bt} + j\cdot \left( {K_{m - 1} \left( a \right) - K_{m} \left( a \right)} \right) + e^{xc} \cdot \sin \left( {2\pi c} \right)\cdot \left( {K_{bt} - K_{m} \left( a \right)} \right)} \\ \end{array} } \right.$$

In this, in the boundary of 0 and 1, the selected arbitrary variable is taken as *c*.

The motion property is enlarged to the n-D space and it is simply shown in Eqs. (4) and (5).

4 $$l_{m}^{p} \left( {a + 1} \right) = \left\{ {\begin{array}{*{20}l} {l_{bt}^{p} \left( a \right) + j\cdot \left( {l_{bt}^{p} \left( a \right) - l_{m}^{p} \left( a \right)} \right) + \beta \cdot \left( {l_{bt}^{p} \left( a \right) - l_{m}^{p} \left( a \right)} \right)} &\quad {m = 1} \\ {l_{bt}^{p} \left( a \right) + j\cdot \left( {l_{m - 1}^{p} \left( a \right) - l_{m}^{p} \left( a \right)} \right) + \beta \cdot \left( {l_{bt}^{p} \left( a \right) - l_{m}^{p} \left( a \right)} \right)} &\quad {m = 2,\ldots ,X} \\ \end{array} } \right.$$

5 $$\beta = 2e^{{j_{1} \frac{A - a + 1}{A}}} \cdot \sin \left( {2\pi j_{1} } \right)$$

In this, the highest iteration is declared by *A* and the weight coefficient is indicated by *β*. The selected arbitrary factor in the range of 0 and 1 is shown as $j_{1}$.

Entire manta rays searching for food sources as its reference region, therefore the cyclone foraging stage attained good exploitation. This strategy enables the MRFO task to gain an effective global search and it is given in Eqs. (6) and (7).

6 $$l_{rn}^{p} = g^{p} + j\cdot \left( {d^{p} - g^{p} } \right)$$

7 $$l_{m}^{p} \left( {a + 1} \right) = \left\{ {\begin{array}{*{20}l} {l_{rn}^{p} \left( a \right) + j\cdot \left( {l_{rn}^{p} \left( a \right) - l_{m}^{p} \left( a \right)} \right) + \beta \cdot \left( {l_{rn}^{p} \left( a \right) - l_{m}^{p} \left( a \right)} \right)} &\quad {m = 1} \\ {l_{rn}^{p} \left( a \right) + j\cdot \left( {l_{m - 1}^{p} \left( a \right) - l_{m}^{p} \left( a \right)} \right) + \beta \cdot \left( {l_{rn}^{p} \left( a \right) - l_{m}^{p} \left( a \right)} \right)} &\quad {m = 2,\ldots ,X} \\ \end{array} } \right.$$

In this, the arbitrary boundary generated arbitrarily in the search area is taken as $l_{rn}^{p}$ and for the *p*th dimension’s upper bound is given by $d^{p}$. For the *p*th dimension’s lower bound is declared by $g^{p}$.

*Somersault foraging* Here, the food’s area is referred to as a pivot. All manta rays swim front and back near the pivot. Hence, the region is updated. This strategy is derived in Eq. (8).

8 $$l_{m}^{p} \left( {a + 1} \right) = l_{m}^{p} \left( a \right) + Z\cdot \left( {j_{2} \cdot l_{bt}^{p} - j_{3} \cdot l_{m}^{p} \left( a \right)} \right),\quad m = 1,\ldots ,X$$

In this, the attribute of somersault is declared as $Z$. It controls the manta ray’s somersault limit. Further, the two arbitrary factors in the range of 0 and 1 are taken as *j*_2_ and* j*_3_. Algorithm 1 elucidates the pseudo-code for MRFO.

**Algorithm 1:**
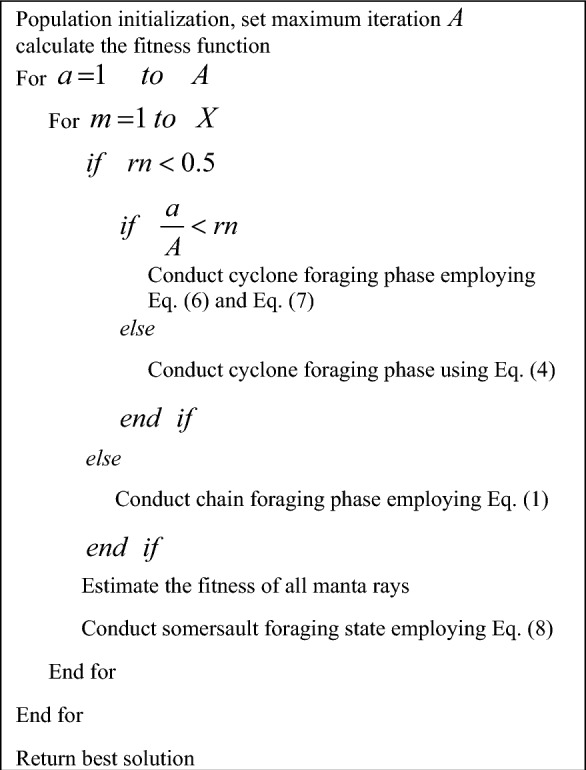
Existing MRFO.

### Conventional algorithm: CBOA

The CBOA^15^ is another meta-heuristic approach by inspiring the strategy of learning the cooking expertise in the training classes. The young and the cooking students are involved in the training classes to improve their cooking expertise and become chefs. This is employed in the CBOA concept.

*Initialization* The CBOA’s candidates are partitioned into two groups “chef instructors and cooking students”. The candidates of CBOA are shown in a matrix based on Eq. (9).

9 $$L = \left[ {\begin{array}{*{20}c} {L_{1} } \\ \vdots \\ {L_{m} } \\ \vdots \\ {L_{X} } \\ \end{array} } \right]_{X \times v} = \left[ {\begin{array}{*{20}c} {l_{1,1} } &\quad \cdots &\quad {l_{1,s} } &\quad \cdots &\quad {l_{1,v} } \\ \vdots &\quad \ddots &\quad \vdots &\quad {\mathinner{\mkern2mu\raise1pt\hbox{.}\mkern2mu \raise4pt\hbox{.}\mkern2mu\raise7pt\hbox{.}\mkern1mu}} &\quad \vdots \\ {l_{m,1} } &\quad \cdots &\quad {l_{m,s} } &\quad \cdots & \quad {l_{m,v} } \\ \vdots &\quad \ddots &\quad \vdots & \quad {\mathinner{\mkern2mu\raise1pt\hbox{.}\mkern2mu \raise4pt\hbox{.}\mkern2mu\raise7pt\hbox{.}\mkern1mu}} &\quad \vdots \\ {l_{X,1} } &\quad \cdots &\quad {l_{X,s} } &\quad \cdots &\quad {l_{X,v} } \\ \end{array} } \right]_{X \times v}$$

The population matrix is specified as *L*. The *m*th candidate of CBOA is denoted as $L_{m}$ and its *s*th dimension is $l_{m,s}$. The size of the population and the issue factor’s count for the objective function are *X* and *s*.

At first, the CBOA’s region is arbitrarily initialized employing Eq. (10).

10 $$l_{m,s} = g_{s} + j\cdot \left( {d_{s} - g_{s} } \right)$$

Here, for the *s*th issue factor’s upper bound is declared by $d_{s}$. For the *s*th issue factor’s lower bound is declared by $g_{s}$. The chosen arbitrary variable among 0 and 1 is specified as *j*.

In CBOA, the objective function is evaluated for each member employing Eq. (11).

11 $$Q = \left[ {\begin{array}{*{20}c} {Q_{1} } \\ \vdots \\ {Q_{m} } \\ \vdots \\ {Q_{X} } \\ \end{array} } \right]_{X \times 1} = \left[ {\begin{array}{*{20}c} {Q\left( {L_{1} } \right)} \\ \vdots \\ {Q\left( {L_{m} } \right)} \\ \vdots \\ {Q\left( {L_{X} } \right)} \\ \end{array} } \right]_{X \times 1}$$

In this, the fitness function’s vector is denoted as *Q*, and the *m*th member’s fitness function is specified as $Q_{m}$.

After initialization, the CBOA moves to enhance the member outcome. The updating task of each group (chef and cooking student) is distinct. The arranged population matrix and fitness function are given in Eqs. (12) and (13).

12 $$LA = \left[ {\begin{array}{*{20}c} {LA_{1} } \\ \vdots \\ {LA_{NC} } \\ {LA_{NC + 1} } \\ \vdots \\ {LS_{X} } \\ \end{array} } \right]_{X \times v} = \left[ {\begin{array}{*{20}c} {la_{1,1} } &\quad \cdots &\quad {la_{1,s} } &\quad \cdots &\quad {la_{1,v} } \\ \vdots &\quad \ddots &\quad \vdots &\quad {\mathinner{\mkern2mu\raise1pt\hbox{.}\mkern2mu \raise4pt\hbox{.}\mkern2mu\raise7pt\hbox{.}\mkern1mu}} &\quad \vdots \\ {la_{NC,1} } &\quad \cdots &\quad {la_{NC,s} } &\quad \cdots &\quad {la_{NC,v} } \\ {la_{NC + 1,1} } &\quad \cdots &\quad {la_{NC + 1,s} } &\quad \cdots &\quad {la_{NC + 1,v} } \\ \vdots &\quad \ddots &\quad \vdots &\quad {\mathinner{\mkern2mu\raise1pt\hbox{.}\mkern2mu \raise4pt\hbox{.}\mkern2mu\raise7pt\hbox{.}\mkern1mu}} &\quad \vdots \\ {la_{X,1} } &\quad \cdots &\quad {la_{X,s} } &\quad \cdots &\quad {la_{X,v} } \\ \end{array} } \right]_{X \times v}$$

13 $$QA = \left[ {\begin{array}{*{20}c} {QA_{1} } \\ \vdots \\ {QA_{NC} } \\ {QA_{NC + 1} } \\ \vdots \\ {qS_{X} } \\ \end{array} } \right]_{X \times v}$$

Here, the chief instructor’s count is given as *NC* and the arranged CBOA’s population matrix is *LA*. The ordered objective function is *QA*. In the matrix *LA*, the candidates from $LA_{1}$ to $LA_{NC}$ define the chef instructors, whereas the candidates from $LA_{NC + 1}$ to $LA_{X}$ define the cooking students.

*Updating the chef instructor group* The chef instructors are highly accountable for instructing the cooking skills to the students who exist in school. In this mechanism, the best instructor is selected and tries to teach the technique to the instructor. According to this, the chef instructor’s region is upgraded by employing Eq. (14).

14 $$la_{m,s}^{C/B1} = la_{m,s} + j\cdot \left( {H_{s} - M\cdot ls_{m,s} } \right)$$

Here, the newly validated status of the arranged *m*th candidate is $la_{m}^{C/B1}$ on the basis of the initial mechanism $C/B1$ and its *s*th dimension is $la_{m,s}^{C/B1}$. The better instructor is provided as *H* and its *s*th dimension is $H_{s}$. From the set {1, 2}, the selected arbitrary variable is *M*. The updated region is accepted only if the fitness value is enhanced. It is derived in Eq. (15).

15 $$LA_{m} = \left\{ {\begin{array}{*{20}l} {LA_{m}^{C/B1} ,} &\quad {QA_{m}^{C/B1} < Q_{m} ;} \\ {LA_{m} } &\quad {else,} \\ \end{array} } \right.$$

Here, the $LA_{m}^{C/B1}$ candidate’s fitness function is taken as $QA_{m}^{C/B1}$.

In the next mechanism, the instructor concentrates to enhance her/his cooking skills on the basis of independent activities. Based on this strategy, for each instructor in the search region, an arbitrary region is produced employing Eqs. (16) to (18). If the arbitrary region enhances the fitness function, it is applicable for position updating employing Eq. (19)

16 $$g_{s}^{lcl} = \frac{{g_{s} }}{a}$$

17 $$d_{s}^{lcl} = \frac{{d_{s} }}{a}$$

18 $$la_{m,s}^{C/B2} = la_{m,s} + g_{s}^{lcl} + j\cdot \left( {d_{s}^{lcl} - g_{s}^{lcl} } \right),\quad m = 1,2,\ldots ,NC,\; j = 1,2,\ldots ,v$$

19 $$LA_{m} = \left\{ {\begin{array}{*{20}l} {LA_{m}^{C/B2} ,} &\quad {QA_{m}^{C/B2} < Q_{m} ;} \\ {LA_{m} } &\quad {else,} \\ \end{array} } \right.$$

Here, the issue factor’s “upper and lower” regions are given as $d_{s}^{lcl}$ and $g_{s}^{lcl}$. The iteration counter is indicated as *a*. Here, the newly validated status of the arranged *m*th candidate is $la_{m}^{C/B2}$ on the basis of the initial mechanism $C/B2$ and its *s*th dimension is $la_{m,s}^{C/B2}$. Here, the $LA_{m}^{C/B2}$ candidate’s fitness function is taken as $QA_{m}^{C/B2}$.

*Updating the cooking student’s group* The students participated in the school to understand the skills of cooking and become chefs. Based on this mechanism, the student is arbitrarily selected in a class taught by the chef. This mechanism estimated the new region utilizing Eq. (20).

20 $$la_{m,s}^{S/B1} = la_{m,s} + j\cdot \left( {K_{{i_{m,s} }} - M\cdot la_{m,s} } \right)$$

Here, the newly validated status of the arranged *m*th candidate is $la_{m}^{S/B1}$ on the basis of the initial mechanism $S/B1$ and its *s*th dimension is $la_{m,s}^{S/B1}$. The chosen chef instructor is provided as $K_{{i_{m,s} }}$ by *m*th the student is $H_{s}$. From the set {1, 2, …, *NC*}, the selected arbitrary variable is $i_{m,s}$.

The updated region is exchanged for the existing region, it enhances the fitness function and it is designed using Eq. (21).

21 $$LA_{m} = \left\{ {\begin{array}{*{20}l} {LA_{m}^{S/B1} ,} &\quad {QA_{m}^{S/B1} < Q_{m} ;} \\ {LA_{m} } &\quad {else,} \\ \end{array} } \right.$$

Here, the $LA_{m}^{S/B1}$ candidate’s fitness function is taken as $QA_{m}^{S/B1}$.

In the second mechanism, each issue factor is considered to be a cooking expertise, and the student concentrates to learn any one chef instructor’s skill entirely. Based on this idea, a new region is estimated by employing Eq. (22).

22 $$la_{m,s}^{S/B2} = \left\{ {\begin{array}{*{20}l} {K_{{i_{m,s} }} ,} &\quad {s = n;} \\ {la_{m,s} ,} &\quad {else,} \\ \end{array} } \right.$$

Further, it is exchanged with the traditional region on the basis of Eq. (23), if it enhances the fitness function.

23 $$LA_{m} = \left\{ {\begin{array}{*{20}l} {LA_{m}^{S/B2} ,} &\quad {QA_{m}^{S/B2} < Q_{m} ;} \\ {LA_{m} } &\quad {else,} \\ \end{array} } \right.$$

Here, the newly validated status of the arranged *m*th candidate is $la_{m}^{S/B2}$ on the basis of the initial mechanism $S/B2$ and its *s*th dimension is $la_{m,s}^{S/B2}$. Here, the $LA_{m}^{S/B2}$ candidate’s fitness function is taken as $QA_{m}^{S/B2}$.

In the third mechanism, each student concentrates to enhance his/her cooking skills on the basis of independent skills. Based on this idea, for each student in the search region, an arbitrary region is produced by Eqs. (16) and (17). Then, a new region is estimated by Eq. (24).

24 $$la_{m,s}^{S/B3} = \left\{ {\begin{array}{*{20}l} {la_{m,s} + g_{s}^{lcl} + j.\left( {d_{s}^{lcl} - g_{s}^{lcl} } \right),} &\quad {s = f;} \\ {la_{m,s} ,} &\quad {s \ne f,} \\ \end{array} } \right.$$

Here, the newly validated status of the arranged *m*th candidate is $la_{m}^{S/B3}$ on the basis of the initial mechanism $S/B3$ and its *s*th dimension is $la_{m,s}^{S/B3}$. The variable *f* is selected from {1, 2, …, v}. Further, it is exchanged with the traditional region on the basis of Eq. (25), if it enhances the fitness function.

25 $$LA_{m} = \left\{ {\begin{array}{*{20}l} {LA_{m}^{S/B3} ,} &\quad {QA_{m}^{S/B3} < Q_{m} ;} \\ {LA_{m} } &\quad {else,} \\ \end{array} } \right.$$

Here, the $LA_{m}^{S/B3}$ candidate’s fitness function is taken as $QA_{m}^{S/B3}$. Algorithm 2 shows the pseudo-code of existing CBOA.

**Algorithm 2:**
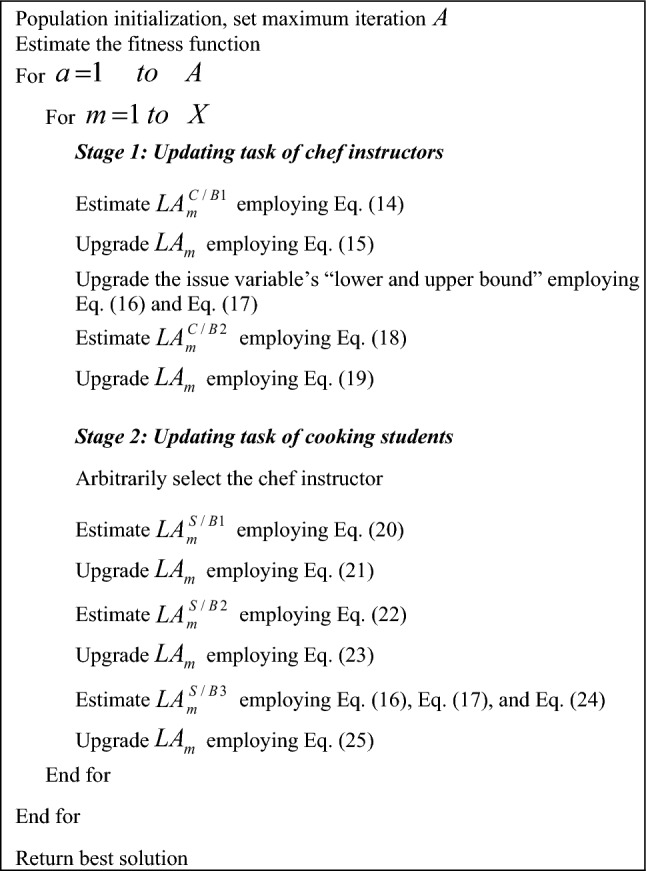
Existing CBOA.

### Proposed hybrid algorithm: HMRFO

The HMRFCO is implemented for optimizing the resource allocation, relay selection, and also the parameters such as steps per epoch, size of epoch, and the count of hidden neurons that exist in the ResGRU technique. Generally, resource allocation faces issues such as poor communication, and resource underutilization. Likewise, the relay selection has several limitations such as inference issues and higher energy consumption. In addition to that, though the deep learning techniques offer promising outcomes, it may face computational complexities. In order to prevent these problems, an effective optimization approach is necessary. Hence, the HMRFCO is introduced for this purpose.

The HMRFCO is the hybrid algorithm, where the CBOA and MRFO algorithms are integrated due to these algorithm’s improved performance rates. The CBOA provides better outcomes for optimization issues and dealing with real-time applications. Similarly, the MRFO can handle complex optimization issues and has better convergence values. However, the CBOA has lower convergence rates, and MRFO struggles to perform well in real time applications. Therefore, these two modern algorithms are integrated and supported in this work. The recommended HMRFCO works on the basis of fitness values and the arbitrary variable in the boundary of 0 and 1. The HMRFCO’s function is mathematically shown in Eq. (26).

26 $$\begin{aligned} &if\; \, j > \frac{crft}{{wrft}}\\ & \qquad \quad Update \quad CBOA \\ & else \\ &\qquad \quad Update \quad MRFO \\ \end{aligned}$$

**Fig. 1:**
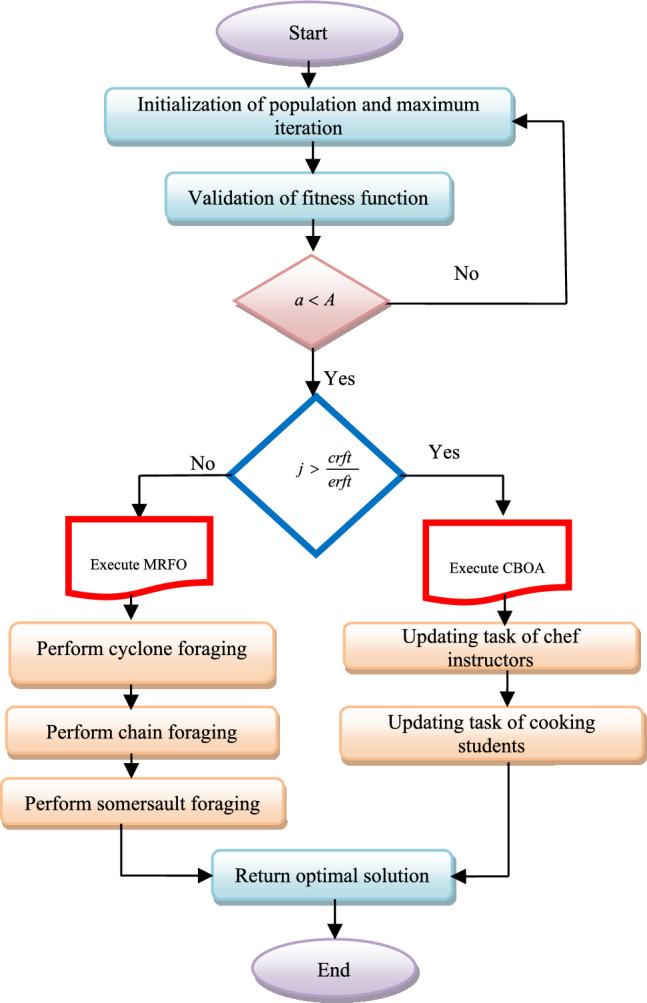
Flowchart of recommended HMRFCO approach for optimization purpose.

Here, the random integer from the limit of 0 and 1 is pointed as *j*, and the worst fitness is declared as *wrft*. The current fitness is given as *crft*. If the selected random integer from 0 to 1 is greater than the value of $\frac{crft}{{wrft}}$ is then the CBOA algorithm is executed or else the MRFO algorithm is executed. Thus, the HMRFCO is implemented for optimization purposes. The pseudo-code of the recommended HMRFCO approach is given in Algorithm 3 and Fig. 1 depicts the HMRFCO approach’s flowchart.

**Algorithm 3:**
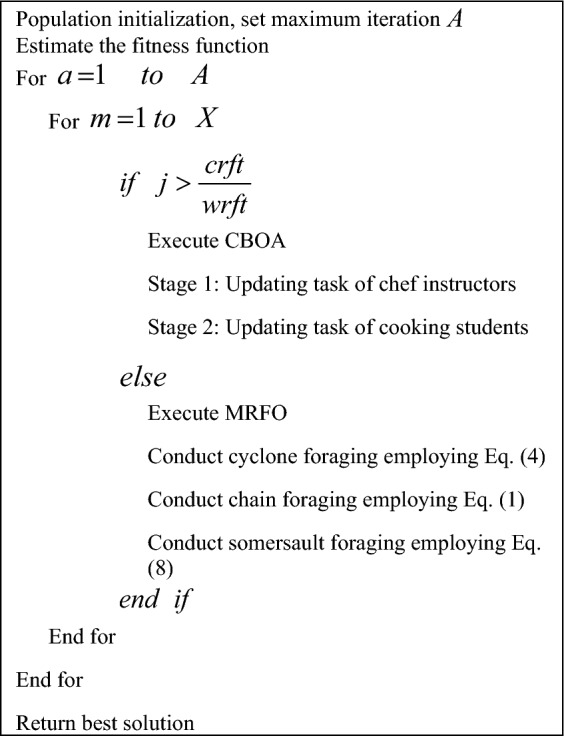
Proposed HMRFCO.

## System model of device-to-device communication and description of sum rate and energy efficiency

### System view of D2D model

**Fig. 2:**
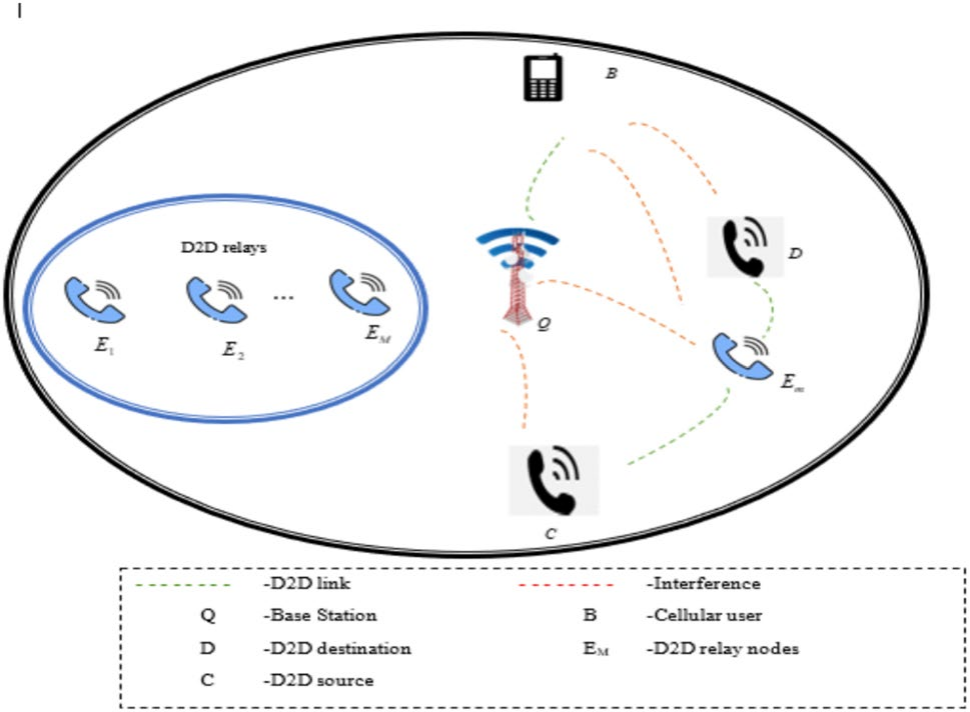
System view of the D2D system.

The system view of the D2D^28^ device is shown in Fig. 2. An independent Cellular user (CU) is presented in the double-layered mobile network and that is denoted as *B* and the destination is referred to as *D*. Moreover, an individual RN is specified as $E_{m} ;\; m = 1,2,\ldots ,M$. An individual source specified as *C* and is taken for this work. The base stations (BS)’s function specified as *Q* is to send power to the candidates of the D2D device on downlink transmission, and to obtain data from CU using the uplink transmission operation. The uplink task is only performed by the BS and it can obtain information via CU. Hence, a “frequency division duplex system” is taken. The allocated resource to the CU *B* concerning the uplink network is reemployed with the aid of the recommended RA-D2D device to one number as maximum. By the spectrum sharing underlay task, the candidates of D2D are enabling to utilize the resources continuously in the cellular uplink network. Hence, the variables $E_{m}$ and $D$ are related to *B*. In the data transmission task, there is an impact on the variable *Q* because of $C$ amd $E_{m}$. In this, it is taken that both the RA-D2D and the *B* connections are offered by the resources from the task of uplink communication. But this task can result in interferences. Hence, reasonable inference handling and power control techniques are necessary to rectify the mutual interference. Thus, the RA-D2D transmission link SINR and the obtained SINR at the BS are desired to rectify the mentioned issues.

The device is considered a “harvest-and-then-transmit”. The overall period desired for the transmission task is segmented into Wireless Energy Transmission (WET) and Wireless Information Transmission (WIT) modules. Further, the WIT is partitioned into two sections, where one sends the information from *C* to the relay at the region *m* in the impact of the interference signal from *B*. The other one sends the information to the relay *m* to *D* in the existence of interference. From the radio frequency (RF) signals, the power is produced by the BS in the WET module. In this protocol, the amplify-and-forward (AF) is employed. In this, a consideration is taken such that always the BS attains the signal achieved from *B* on the module of WIT. The power of the signal $K_{0}$ is more than the noise power in the WET module as the D2D systems attain the signals of RF from the BS. Hence, no power is generated from the noise that exists in the channels. The energy produced in the WET module is formulated in Eqs. (27), (28), and (29).

27 $$W_{C} = l_{0} \eta K_{0} e_{Q,C}$$

28 $$W_{{D_{m} }} = l_{0} \eta K_{0} e_{{Q,C_{m} }}$$

29 $$W_{D} = l_{0} \eta K_{0} e_{Q,C}$$

In the above expressions, the variable $\eta \in \left( {0,1} \right)$ refers to the energy conversion’s efficacy. The variable $l_{0}$ indicates the time utilized for the WET module and the energy gain is referred to as *e*. The SINR γ of data transferred from $C$ to $E_{m}$ in the WIT stage is expressed in Eqs. (30) and (31).

30 $$\gamma_{{C,E_{m} }} = \frac{{K_{C} E_{{C,E_{m} }} }}{{K_{B} e_{{B,E_{m} }} + X_{0} }}$$

31 $$\gamma_{{E_{m} ,D}} = \frac{{K_{{E_{m} }} e_{{E_{m} ,D}} }}{{K_{B} e_{B,D} + X_{0} }}$$

Here, the noise variance is specified as $X_{0}$.

The joint achieved SINR at *D* is shown in Eq. (32).

32 $$\gamma_{{CCEE_{mm} ,DD}} = \min \left\{ {\gamma_{{CE_{l} }} ,\gamma_{{E_{m} D}} } \right\}$$

The data rate of the RA-D2D connection is given in Eq. (33).

33 $$EE_{{CE_{m} DC}} = l_{c} L\log_{2} \left( {1 + \gamma_{{CE_{m} D}} } \right);\quad d = 1,2$$

Here, the $L$ specifies the bandwidth (fixed) that is offered in the system. The network faced two interferences one was data transmission from $C$ to $,E_{m}$ and the other was data transmission from $E_{m}$ to $D$. Hence, the BS attained SINR as derived in Eq. (34).

34 $$\gamma_{B,Q} = \left\{ {\begin{array}{*{20}l} {\frac{{K_{B} e_{B,Q} }}{{K_{{E_{m} }} e_{{Q,E_{m} }} + X_{0} }}} &\quad {at\; l_{2} } \\ {\frac{{K_{B} e_{B,Q} }}{{K_{C} e_{Q,C} + X_{0} }}} &\quad {at \; l_{1} } \\ \end{array} } \right.$$

The information rate is formulated in Eq. (35)

35 $$E_{Y} = l_{d} L\log_{2} \left( {1 + \gamma_{B,Q} } \right);\quad d = 1,2$$

The overall energy employed in this module is shown in Eq. (36).

36 $$W = l_{1} \frac{{K_{C} }}{\beta } + l_{2} \frac{{K_{{E_{m} }} }}{\beta }$$

Here, the variable $\beta \in \left( {0,1} \right)$ specifies the power amplifier’s efficacy. The attributes $l_{1}$ and $l_{2}$ are the time employed for the WIT module.

### Definition of sum rate

The variable $SE\left( {Y,L} \right)$ refers to the sum rate in that, the attribute *Y* specifies the matrix illustration of the variable $g_{v,b}$. In addition, the matrix illustration of the variable *L* is indicated as $s_{v,b}$. Equation (37) derives the sum rate estimation.

37 $$SE\left( {Y,L} \right) = \sum\limits_{v \in D} {\sum\limits_{b \in X} {g_{v,b} SE_{b} \left( {g_{v,b} ,s_{v,b} } \right)} }$$

The pairs of the D2D system are given as $X_{v}$ and this pair of D2D $X_{v}$ distributes the resource equal to the variable. The variable’s $X_{v}$ value is hence offered as $X_{v} = \left\{ {b|g_{v,b} = 1,\forall b \in X} \right\}$. The communication device of D2D’s sum rate is concentrated to maintain at highest value. Equation (38) offers the mathematical format of this objective.

38 $$ob_{1} = \mathop {\arg \,\min }\limits_{{\left\{ {g_{v,b} ,s_{v,b} } \right\}}} \left( {\frac{1}{{SE\left( {Y,L} \right)}}} \right)$$

The upcoming criteria are employed for achieving the above objective. To make the D2D pair connect with the individual CU in the resource module, the factor is employed ${g}_{v,b},{s}_{v,b}\in \left\{0,1 \right\},$ $\forall v\in D;b\in X$.

The CU is assigned to most individual D2D pairs by the factor’s utilization $\sum\nolimits_{e \in U} {g_{v,b} \le 1,\; \forall b \in X}$ and $\sum\nolimits_{{bX_{v} }} {s_{v,b} \le 1,\; \forall v \in D}$ such that $s_{v,b} = 0,\; \forall b \notin X_{v} ;v \in D$.

The needed rate by each D2D pair is defined in terms of factor $SE_{b} \left( {g_{v,b} ,s_{v,b} } \right) \ge S\overline{E}_{b}$.

In the entire optimization issue, there are no concave features are displayed toward the factors $g_{v,b}$ or $s_{v,b}$. Hence, the above issue is considered a binary integer, non-linear, and non-convex issue with the 2*DX* attributes. The factor is reduced to an “NP-hard issue or 0–1 Knapsack issue”. The goal of achieving a high sum rate is accomplished by tuning the resource allocated $OP_{op}^{FC}$ in the D2D system and transmission power $TR_{tr}^{FC}$ by employing the HMRFCO approach to optimally choose the relay amounts along with conducting the joint resource allocation in the D2D system.

### Definition of energy efficiency

The ratio among the data rate in the AR-D2D and the energy utilized by the D2D device is explained by the variable in this process. Equation (39) derived the EE.

39 $$EE = \frac{{G_{{CE_{m} ,I}} }}{j}$$

Here, the data rate is indicated as *G*, and the utilized energy is taken as *j*. The D2D system’s destination is given as *I* and the D2D system’s source is declared as *C*. The D2D system’s relay is specified as $E_{m}$.

Enhancing the EE in the D2D transmission device by referring to the rate of data, SINR attained at *E*, time distribution *l*, and power control *K* is the primary objective of the optimal relay selection task. Equation (40) shows the objective of this task.

40 $$ob_{2} = \mathop {\arg \,\,\,\min }\limits_{{\left\{ {l,K_{k} ,K_{{E_{m} }} } \right\}}} \left( \frac{1}{EE} \right) = \frac{{l_{1} K_{C} + l_{2} K_{{E_{mm} }} }}{{G_{{CE_{m} ,I}} }}$$

Here, the variable $C\cdot l\cdot j_{1} :0 < l_{1} K_{C} \le J_{k} ,0 < l_{2} K_{{E_{m} }} \le J_{{E_{m} }}$. The variable $j_{1}$ specifies the utilized energy by the D2D system in the WIT module. The variable’s $j_{1}$ value must not become higher than the energy that is produced in the WER module of the D2D device. The attributes that are needed to fulfill the lower SINR are denoted as $j_{2}$ and $j_{3}$.

These attributes are in the boundary presented as $j_{2} \to \left( {\gamma_{{CE_{m} }} \ge \gamma_{{E_{m} }}^{\min } } \right)$ and $j_{3} \to \left( {\gamma_{{E_{m} }} \ge \gamma_{I}^{\min } } \right)$. The boundary provided to the information rate that has to be attained with the aid of DS in the D2D transmission device is specified as $j_{4}$. The variable’s $j_{4}$ value is in the boundary $j_{4} \to \left[ {L \log_{2} \left( {1 + \gamma_{B,Q} } \right) \ge E_{B,Q}^{\min } } \right]$. This boundary allotment for the data transmission is indicated as $j_{5}$. The variable’s $j_{5}$ value exists in the bound $l_{2} + l_{0} + l_{1} \le 1$. The non-negative limit set to the assigned time and power is indicated as $j_{6}$. The variable’s $j_{6}$ value is in $l_{2} + l_{0} + l_{1} ,K_{CC} ,K_{{E_{m} }} > 1$. The objective $ob_{2}$ is neither convex nor concave. Hence, fractional programming is employed to transmit it to an effective form. The goal of achieving higher EE is obtained with the transmission power $TR_{tr}^{FC}$ optimization in the D2D system by employing the recommended HMRFCO to optimally choose the relays in the D2D system.

## Forecasting the resource and relay in D2D model using residual GRU and multi-objective function

### Adaptive residual GRU for prediction

The AResGRU technique is supported in this work for performing the joint prediction. The ResGRU^44^ is an effective and recent deep learning approach. The ResNet can rectify the issue of system functionality degradation and the non-convergence issue created by the depth of the network. In the framework of the residual accumulation layer, it is considered that the input *y* is and the features learned with the aid of the network are denoted as $I\left( y \right)$. It is expected that the network can understand the residual $G\left( y \right) = I\left( y \right) - y$, hence that the network’s real learning feature is enhanced to $G\left( y \right) + y$. The accumulation layer performs the identity mapping when the residual value is zero to prevent the redundancy created by the layer of the redundant network. The gradient is also rectified by this network. But, practically, the residual is not equal to zero. It makes the accumulation layer learn the new attributes of the given features, hence having better functionality.

A normal GRU can rectify the issue of gradient explosion effectively. But, when the input amount gets enhanced, the GRU causes network degradation, leading to the loss of several features of input data. To rectify this issue, a ResGRU is introduced. The GRU approach is employed in the residual block to draw out the time series features. In the ResGRU network, the residual module’s outcome is identical to the total of the GRU technique’s last layer’s outcome and the input *y*. Considering that the final layer of the GRU is $z$ and the outcome $z_{R}$ of the residual module is derived in Eq. (41).

41 $$z_{R} = {\text{Re}} LU\left( {BN_{\alpha ,\beta } \left( z \right) + h\left( {y_{f} } \right)} \right)$$

Here, ${\text{Re}} LU\left( \cdot \right)$ is the activation function, and the batch normalization is indicated as $BN\left( \cdot \right)$. In the function, the two learnable attributes are given as $\alpha$ and $\beta$. The adjustment function is denoted as $h\left( \cdot \right)$, making $y_{f}$ and $i_{f}$ with equal dimensions. Through the residual link, the ResGRU technique can remember the correlation among the data after and before the data and enhance the prediction functionality of the network while retaining the original data’s characteristic information.

*AResGRU* As mentioned earlier, the ResGRU is employed for performing the joint prediction task. However, the ResGRU can face computational burdens because of the network parameter’s high count. Hence, optimizing the network parameters such as steps per epoch, size of epoch, and the counts of hidden neurons are very significant in the prediction task. Because of this optimization process, the system errors are also reduced. For this objective, the HMRFCO is recommended. This is a hybrid algorithm with effective functionalities. Based on this algorithm, the mentioned network parameters are optimized. Equation (42) offers the objective function of this operation.

42 $$ob_{3} = \mathop {\arg \,\,\,\min }\limits_{{\left\{ {hn^{{{\text{Re}} sGRU}} ,ep^{{{\text{Re}} sGRU}} ,se^{{{\text{Re}} sGRU}} } \right\}}} \left[ {RMSE + MSE + MAE} \right]$$

Here, the ResGRU’s hidden neuron count is $hn^{{{\text{Re}} sGRU}}$ and varies from [5–255]. The ResGRU’s epoch size is $ep^{{{\text{Re}} sGRU}}$ and varies from [5–50]. The ResGRU’s steps per epoch count are $se^{{{\text{Re}} sGRU}}$ and vary from [5–50]. In addition to that, the “root mean square error (*RMSE*), mean square error (*MSE*), and mean absolute error (*MAE*)” are minimized in the network by the suggested HMRFCO. These performance metrics are explained as follows.

*RMSE:* It is the performance signal that validates the difference among the actual and predicted values. It is expressed in Eq. (43)

43 $$RMSE = \sqrt {\frac{{\sum\nolimits_{h = 1}^{V} {\left\| {s\left( f \right) - \overset{\lower0.5em\hbox{$\smash{\scriptscriptstyle\frown}$}}{s} \left( f \right)} \right\|^{2} } }}{V}}$$

*MSE:* It is a statistical factor of how effectively an estimator works. It is formulated in Eq. (44).

44 $$MSE = \frac{1}{V}\sum\limits_{h = 1}^{V} {\left( {s\left( f \right) - \overset{\lower0.5em\hbox{$\smash{\scriptscriptstyle\frown}$}}{s} \left( f \right)} \right)^{2} }$$

*MAE:* It is the factor of the mean size of faults in a group of predictions without considering directly. It is derived in Eq. (45).

45 $$MAE = \frac{{\sum\nolimits_{h = 1}^{V} {\left| {s\left( f \right) - \overset{\lower0.5em\hbox{$\smash{\scriptscriptstyle\frown}$}}{s} \left( f \right)} \right|} }}{V}$$

**Fig. 3:**
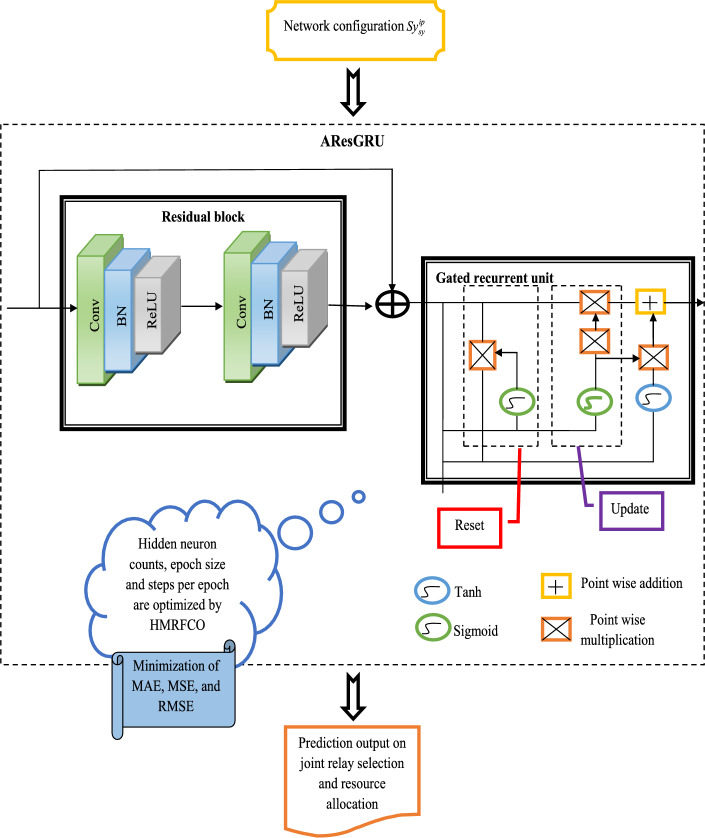
Structural diagram of recommended AResGRU for prediction.

Here, the amount of data points is denoted as $$V$$. The variable $$s\left( f \right)$$ is the *f*th measurement and its related prediction is specified as $$\overset{\lower0.5em\hbox{$\smash{\scriptscriptstyle\frown}$}}{s} \left( f \right)$$. Thus, the AResGRU is constructed for the prediction purpose using the HMRFCO approach. Here, the attributes such as “network size, number of D2D relays, mobility static, D2D source coordinates, the distance between D2D users, cellular user coordinates, cellular user base station coordinates, bandwidth, D2D destination coordinates, minimum decodable SINR at the D2D relay, minimum data rate requirement of the cellular user, minimum decodable SINR at the D2D destination, power amplifier efficiency, noise spectral density, the transmission power of the BS, the power conversion efficiency, the transmission power of the cellular user, and path gain for each link” are employed as the primary system configurations that are employed as input for the suggested optimal resource allocation and joint relay selection. These significant attributes are denoted as $$Sy_{sy}^{ip}$$ and are given as input for the designed AResGRU network for making predictions. Finally, an accurate predicted outcome is achieved by the designed AResGRU approach and its structural diagram is shown in Fig. 3.

### Joint approach of resource and relay selection using HMRFCO

Optimal relay selection and resource allocation are significant concepts in the D2D communication system. Resource allocation is the task of detecting and assigning the resources to the network to support the data transmission. Similarly, the relay selection is the operation of selecting the delay to better system reliability and performance. However, these two tasks may face limitations such as high-power consumption, interferences, and so on. These limitations must be resolved to perform error-free data transmission. Therefore, the attributes such as allocated resources for the channel and the transmission power must be optimized. For this objective, the suggested HMRFCO is supported due to its better functionalities. These attributes are optimally tuned to select the optimal number of relays for performing the data transmission in the network.

**Fig. 4:**
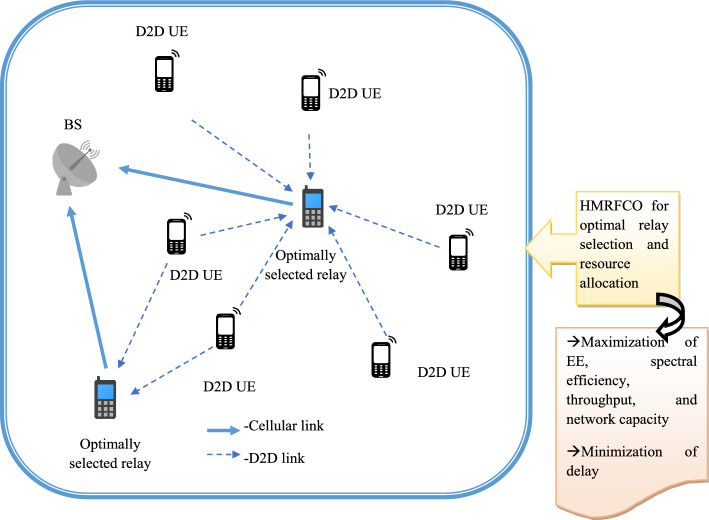
Pictorial representation of HMRFCO-based optimal resource allocation and relay selection.

The transmission power optimization supports to improve the network lifespan and assists to manage the device connectivity in the D2D system. This is also supporting to minimize the energy consumption. Therefore, the network EE is increased. Moreover, to improve the spectral efficacy without affecting the Quality of Service (QoS), the D2D system resources have to be selected optimally. The improvement in spectral efficiency is supportive of minimizing the SNR and improving the capacity of the channel to perform the data transmission. The pictorial representation of HMRFCO-based optimal resource allocation and relay selection is given in Fig. 4.

### Derivation of multi-objective function

As explained before, the HMRFCO is utilized for optimizing the allocated resources for the channel and the transmission power. By this process, the network complexities are prevented and the network’s efficiency is improved. This maximizes the network EE, spectral efficiency, network capacity, and throughput. Moreover, it minimizes the network delay. The objective function of this process is given in Eq. (46).

46 $$ob_{4} = \mathop {\arg \,\,\,\,\max }\limits_{{\left\{ {TR_{tr}^{FC} ,OP_{op}^{FC} } \right\}}} \left[ {SE + EE + TP + NC + \frac{1}{DY}} \right]$$

Here, the optimized transmission power is indicated as $TR_{tr}^{FC}$, which varies from [2–128]. Further, the optimally selected resource is specified as$OP_{op}^{FC}$ it ranges from [1-no of the channel]. Further, the network EE, spectral efficiency, network capacity, delay, and throughput are indicated as *EE*, *SE*, *NC*, *DY*, and *TP* respectively.

## Results and discussions

### Simulation setup

The implemented joint optimal relay selection and the resource allocation mechanism for the D2D communication device were implemented by employing the platform named MATLAB 2020a. Here, the HMRFCO approach utilized 10 populations, 250 highest iterations, and 3 as chromosome length. The synthetic simulation was used to generate dataset based on 3GPP TR 38.901 Urban Macrocell standard channel model by varying parameters. It includes relay node, mobility, noise, resource allocation, and interference, etc. The dataset includes 20,000 samples, split into 80% for training and 20% for testing of the AresGRU model across variable node densities (50–500), sub-channels (1–5), and relay positions. Hyperparameters were optimized by using the proposed HMRFCO algorithm to achieve minimal prediction error. Simulation and system parameter values and ranges have been mentioned in the Tables 2 and 3. The offered mechanism’s performance was validated by comparing it with conventional algorithms and techniques such as “Sand Cat Swarm Optimization (SCO)^45^, Flow Direction Algorithm (FDA)^46^, MRFO^47^, CBOA^9^, TRSPC^20^, RPRS-EH^21^, Multi-relay system model^22^, and EHA-CRD^28^”. Moreover, some of the conventional prediction techniques such as “deep neural network (DNN)^48^, support vector machine (SVM)^49^, long short-term memory (LSTM)^30^, ResGRU^44^ and and hybrid flow direction with chameleon swarm algorithm-adaptive multi-layer perceptron (HFDCSA-AMLP)^41^” were employed for analyzing the recommended prediction approach.

**Table 2:** System parameters.

Parameter	Description
Cell size	500 m × 500 m
Nodes	[50, 100, 150, 200, 250]
Mobility	Static
Number of CUEs	10–20
Number of DUEs	15–30
Number of relays	5–25
Number of resource blocks	1–5
Carrier frequency	2.1 GHz
Bandwidth per RB	180 kHz
Minimum data rate requirements	20–30 kbps
Transmission power (BS)	45–55 dBm
Transmission power (D2D)	23 dBm or 0.2 W
Transmission power (Relay)	20 dBm or 0.1 W
SINR threshold	5 dB
Channel model	Rayleigh fading + Log-normal shadowing
Pathloss model	3GPP urban macrocell (TR 38.901)
Noise power density	− 174 dBm/Hz
Mobility model	Random waypoint model

**Table 3:** Simulation and model parameters.

Parameter	Description
Simulation tool	MATLAB R2020a
Number of simulation runs	30
Random seed	42
HMRFCO population size	10
HMRFCO max iterations	250
Chromosome length	3(sun-channel selection, power allocation, relay index)
HMRFCO convergence threshold	1e−5
AResGRU optimizer	Adam
Learning rate	0.001
Batch size	32 [5–50]
Epochs	25 [5–50]
Hidden layers (AResGRU)	2 GRU layers [128 and 64 neurons] [5–255]
Activation function	ReLU
Loss function	Mean square error (MSE)
Evaluation metrics	EE, Delay, Throughput, RMSE, MAE, Success rate

### Convergence evaluation of recommended HMRFCO

**Fig. 5:**
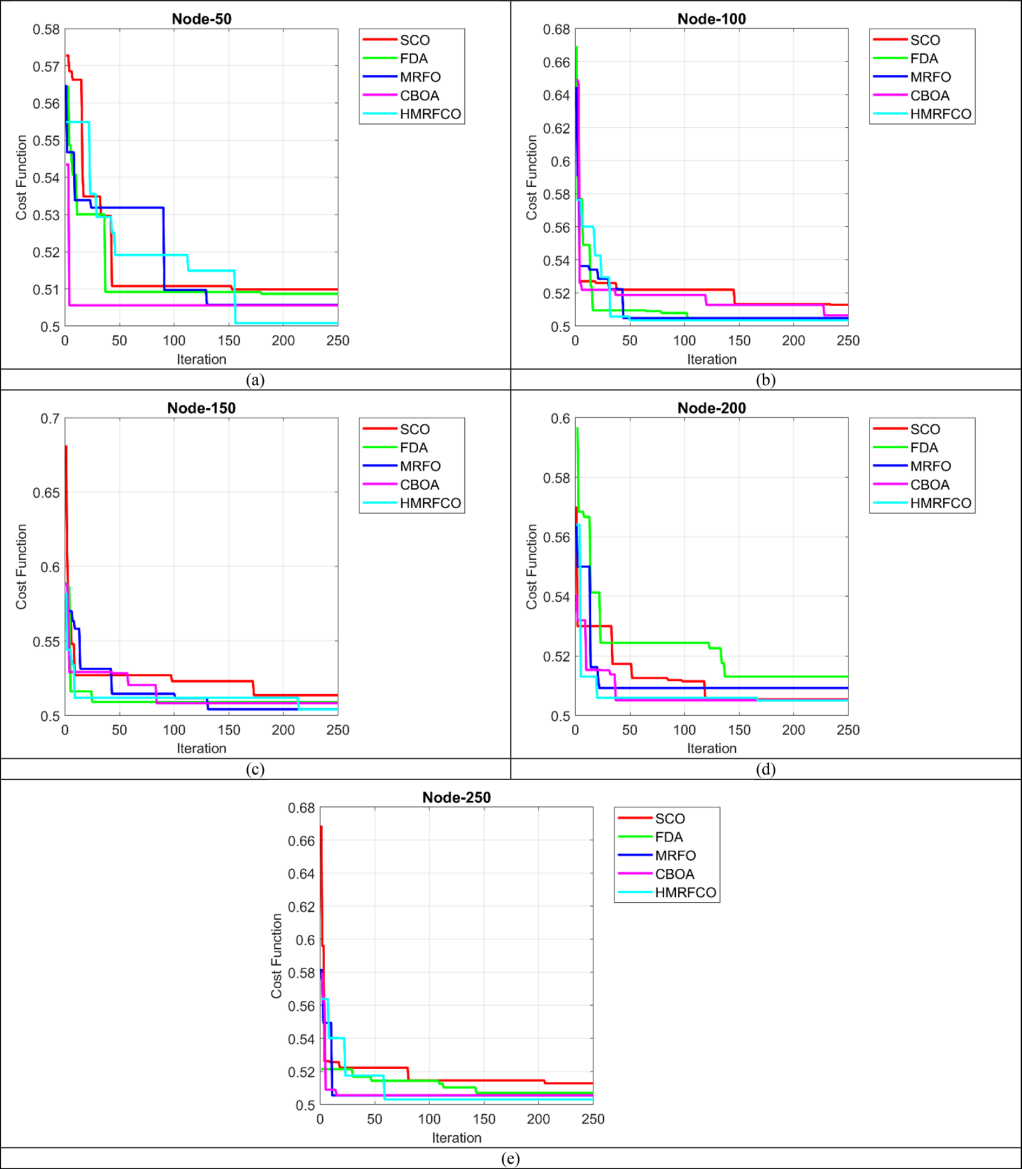
Convergence validation of suggested HMRFCO approach over traditional optimization algorithms in terms of “(**a**) Node-50, (**b**) Node-100, (**c**) Node-150, (**d**) Node-200, and (**e**) Node-250”.

The recommended HMRFCO approach’s convergence assessment is carried out in Fig. 5 over various conventional optimization algorithms with the support of iteration counts. By varying the iteration counts, the convergence assessment is performed for the nodes 50, 100, 150, 200, and 250 in Fig. 5a–e, respectively. This evaluation has helped to validate the performance rates of the presented HMRFCO approach over conventional algorithms. When taking the 200th iteration in Fig. 5a, it has been shown that the suggested HMRFCO attained 2%, 1.8%, 1%, and 1% higher convergence rates than the traditional algorithms such as SCO, FDA, MRFO, and CBOA respectively. This assessment has shown that, for all nodes the recommended HMRFCO achieving 95% of the optimal fitness within 180 iterations, but MRFO required 208 iterations, which is 13.35% faster than MRFO. Thus, it has been ensured that the presented HMRFCO approach is highly suitable for the recommended joint optimal resource allocation and relay selection mechanism in the D2D communication model.

### Statistical evaluation of recommended HMRFCO

The statistical validation of the presented HMRFCO is carried out over conventional optimization approaches and presented in Tables 4 and 5. Here, the statistical metrics including “best, worst, mean, median, and standard deviation” are considered in this evaluation. This evaluation supports to validate whether the presented HMRFCO approach can able to choose the optimal relay, parameters, and resources for the D2D communication system. When considering the worst factor in the 250th node, the designed HMRFCO approach attained 17.97%, 8.01%, 2.67%, and 2.19% better solutions than the traditional algorithms such as SCO, FDA, MRFO, and CBOA accordingly. These findings show that the suggested HMRFCO approach is more effective than the classical algorithms for achieving optimal solutions. For other node values also the presented HMRFCO showed better outcomes.

**Table 4:** Statistical significance of the proposed method.

Metric	Compared methods	Mean difference	*p* value	95% confidence interval	Test used
Energy efficiency (EE) (bits/joule)	HMRFCO vs. MRFO	+ 0.043	0.0031	[0.0031, 0.0058]	Paired t-test
Delay (ms)	HMRFCO vs. MRFO	− 4.3	0.0125	[− 6.8, − 1.1]	Wilcoxon signed rank
Throughput (Mbps)	HMRFCO vs. TRSPC	+ 6.3	0.0018	[4.0, 8.7]	Paired t-test
RMSE	AResGRU vs. ResGRU	− 6.98	0.0028	[− 9.5. − 3.1]	Paired t-test

**Table 5:** Statistical evaluation of recommended HMRFCO algorithm over traditional optimization approaches.

Terms	SCO^45^	FDA^46^	MRFO^47^	CBOA^50^	HMRFCO
“Node-50”					
“Best”	50.985	50.866	50.571	50.558	50.077
“Worst”	57.276	56.437	56.485	54.347	55.486
“Mean”	51.625	51.283	51.642	50.604	51.56
“Median”	51.075	50.917	50.974	50.558	51.49
“Standard deviation”	1.4938	1.0115	1.343	0.41332	1.5646
“Node-100”					
“Best”	51.292	50.352	50.49	50.641	50.348
“Worst”	59.561	66.946	64.472	64.889	57.63
“Mean”	51.978	50.902	51.007	51.703	50.959
“Median”	52.201	50.352	50.49	51.277	50.348
“Standard deviation”	0.98083	1.6165	1.4594	1.5156	1.6809
“Node-150”					
“Best”	51.361	50.899	50.42	50.829	50.413
“Worst”	68.131	58.942	57.023	58.846	58.216
“Mean”	52.341	51.081	51.381	51.492	51.184
“Median”	52.309	50.899	51.177	50.829	51.191
“Standard deviation”	1.4128	0.99278	1.4759	1.146	0.72187
“Node-200”					
“Best”	50.538	51.31	50.922	50.513	50.499
“Worst”	57.017	59.653	56.433	54.062	56.4
“Mean”	51.146	52.218	51.16	50.72	50.697
“Median”	50.538	52.261	50.922	50.513	50.593
“Standard deviation”	0.89678	1.3722	0.93672	0.58812	0.75118
“Node-250”					
“Best”	51.282	50.711	50.552	50.574	50.306
“Worst”	66.883	52.149	58.211	57.939	56.393
“Mean”	51.817	51.175	50.75	50.691	50.909
“Median”	51.468	51.046	50.552	50.574	50.306
“Standard deviation”	1.2612	0.4919	1.0008	0.82305	1.3473

The following statistical analysis was carried out to guarantee the reliability and validity of the results obtained:

Each simulation was repeated 30 times with different random initializations to account for randomness in optimization and prediction processes.

For each performance metric (EE, Delay, Throughput, and RMSE), we calculated the mean, median, best value, worst value, and standard deviation over the 30 times.

To validate whether the proposed method (HMRFCO and AresGRU) was significantly outperformed by baseline methods.

We have checked the normality of the data distribution. If the data were normally distributed—a paired t-test was used (parametric test). Energy efficiency and RMSE are used in a paired t-test.

If the data was not normally distributed, a Wilcoxon signed rank test (non-parametric test) was used. Delay is not normally distributed; a Wilcoxon test was used. For each comparison, 95% confidence intervals were calculated.

Therefore, the suggested HMRFCO for relay selection and resource allocation was confirmed by its consistent statistical superiority (*p* < 0.05) across all the node densities.

### Number of nodes-based performance estimation of suggested joint optimal resource allocation and relay selection mechanism in the D2D communication system

**Fig. 6:**
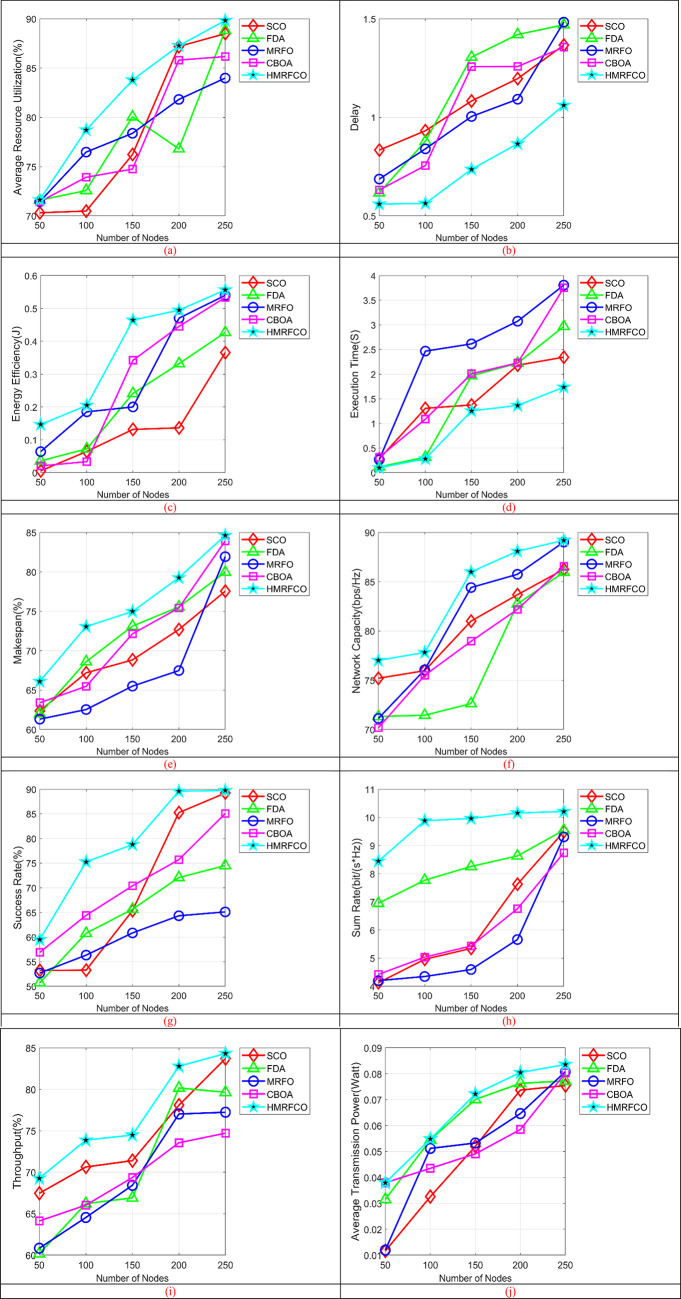
Number of nodes-based performance analysis of recommended joint optimal relay selection and resource allocation mechanism over traditional algorithms concerning “(**a**) Average resource utilization, (**b**) Delay, (**c**) Energy efficiency, (**d**) Execution time, (**e**) Makespan, (**f**) Network capacity, (**g**) Success rate, (**h**) Sum rate, (**i**) Throughput”, and (**j**) Average transmission power”.

**Fig. 7:**
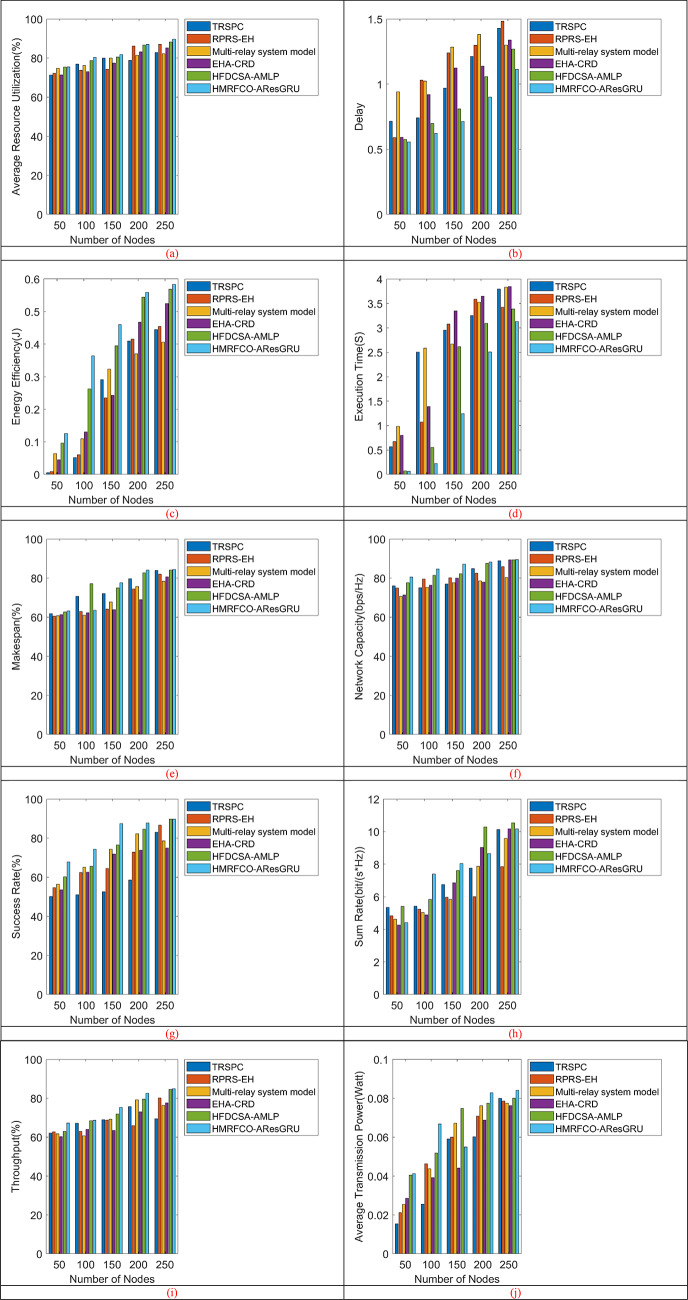
Number of nodes-based performance analysis of recommended joint optimal relay selection and resource allocation mechanism over traditional techniques concerning “(**a**) Average resource utilization, (**b**) Delay, (**c**) Energy efficiency, (**d**) Execution time, (**e**) Makespan, (**f**) Network capacity, (**g**) Success rate, (**h**) Sum rate, (**i**) Throughput”, and (**j**) Average transmission power”.

The performance of the recommended approach is evaluated over conventional optimization algorithms and techniques by varying the number of nodes. Figure 6 shows the number of nodes-based performance validations of the recommended approach with relay over conventional optimization algorithms. Figure 6a shows that HMRFCO achieves superior resource utilisation by allocating power and channels. Figure 6b indicates delay, Fig. 6c depicts the energy efficiency, Fig. 6d shows the execution time, Fig. 6e represents the make span, Fig. 6f–j explore the network capacity, success rate, sum rate, throughput and average transmission power, respectively. Figure 7 (Fig. 7a–j, same as Fig. 6) depicts the number of nodes-based performance validations of the recommended approach with relay over conventional techniques. This evaluation has shown that the presented work is capable of working better with and without relays than the conventional algorithms and techniques. When considering the relay-based analysis, the energy efficiency of the presented scheme is increased by 40%, 27.27%, 1.81%, and 3.63% higher than the conventional algorithms such as SCO, FDA, MRFO, and CBOA accordingly in Fig. 6c for 250th node. Moreover, it has been shown that the recommended scheme attained an energy efficiency of 0.764 bps/J, which is 5.95% more energy efficiency than the traditional algorithms. Also, it is same for the method analysis. When considering the without relay-based analysis, the suggested scheme’s throughput is highly enhanced by 33.82%, 19.11%, 8.23%, 19.11%, and 4.41% than the traditional techniques such as TRSPC, RPRS-EH, Multi-relay system model, EHA-CRD, and HFDCSA-AMLP appropriately in Fig. 7i for 200th node. This analysis has guaranteed that the suggested approach achieved 33.82% higher throughput than TRSPC, if there is no relay. Moreover, it has shown that the presented work attained 8.14% reducing delay than the conventional models. This is similar to the algorithm-based experiments also.

### Number of subchannels based performance estimation of suggested joint optimal resource allocation and relay selection mechanism in the D2D communication system

**Fig. 8:**
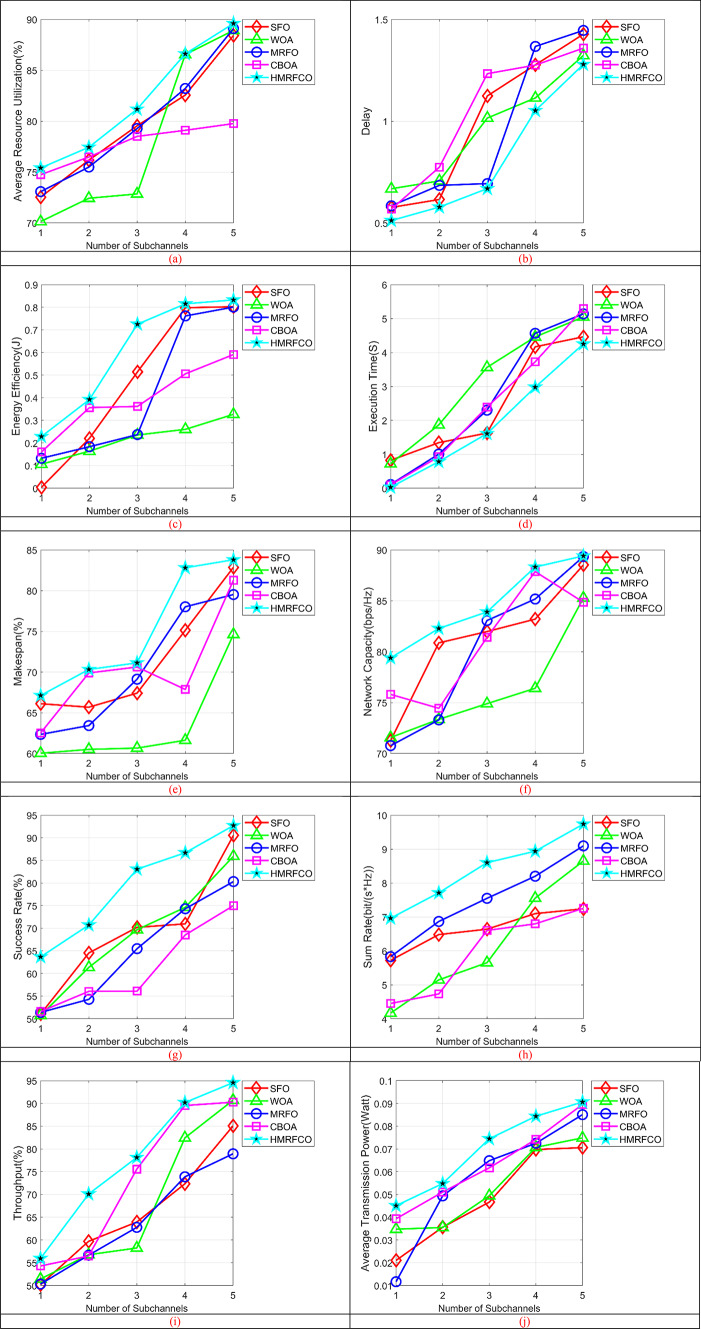
Number of subchannels-based performance analysis of recommended joint optimal relay selection and resource allocation mechanism over traditional algorithms concerning “(**a**) Average resource utilization, (**b**) Delay, (**c**) Energy efficiency, (**d**) Execution time, (**e**) Makespan, (**f**) Network capacity, (**g**) Success rate, (**h**) Sum rate, (**i**) Throughput, and (**j**) Average transmission power”.

**Fig. 9:**
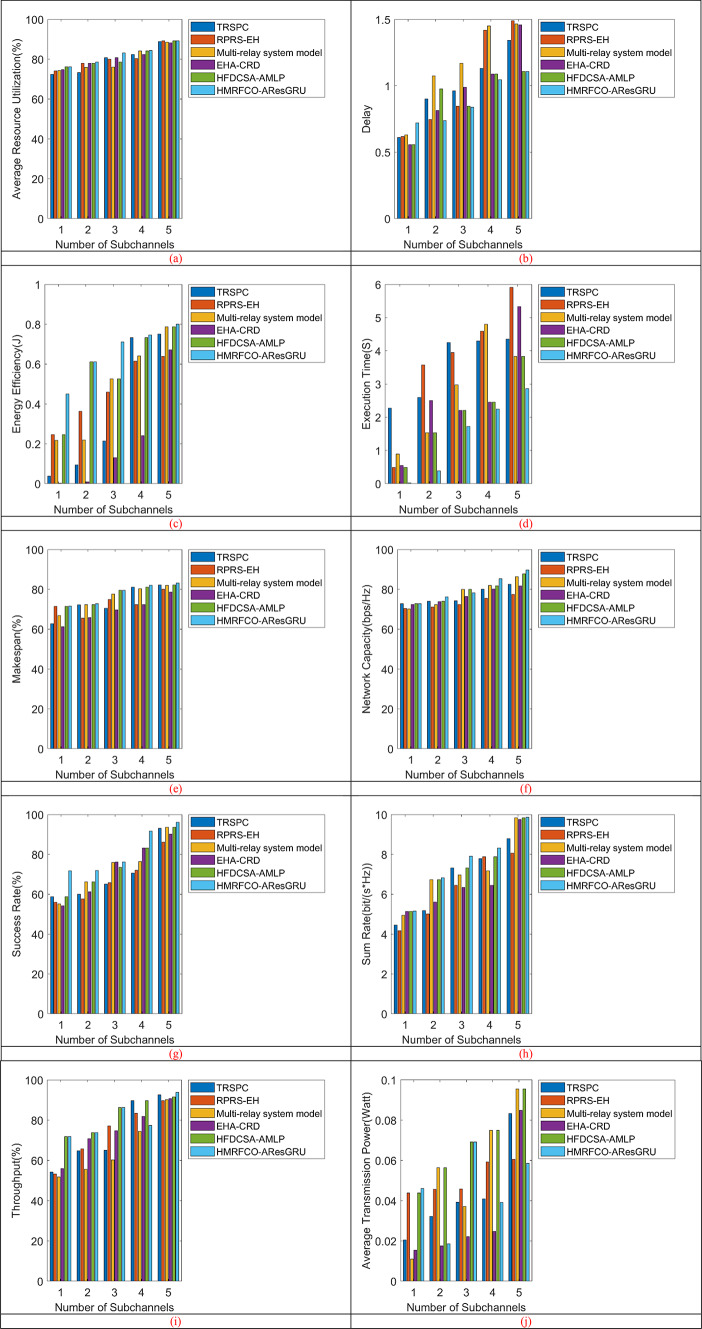
Number of subchannels-based performance analysis of recommended joint optimal relay selection and resource allocation mechanism over traditional techniques concerning “(**a**) Average resource utilization, (**b**) Delay, (**c**) Energy efficiency, (**d**) Execution time, (**e**) Makespan, (**f**) Network capacity, (**g**) Success rate, (**h**) Sum rate, (**i**) Throughput, and (**j**) Average transmission power”.

The performance of the recommended joint optimal relay selection and resource allocation mechanism’s performance is analyzed using the various numbers of sub-channels over existing optimization techniques and techniques. Figure 8 displays the number of sub-channel-based performance analyses of recommended mechanisms with and without relays over conventional optimization approaches. Subgraphs from Fig. 8a–j show that the HMRFCO will gain with an increase in the number of subchannels, delay and makespan were decreased, network capacity, success rate were maximized, and adaptive power control also efficiently reduces the transmission. Figure 9 (Subgraphs from Fig. 9a–j) shows the number of sub-channel-based performance analyses of recommended mechanisms with and without relays over conventional techniques. When considering the relay-based analysis, it has been shown that the recommended scheme’s delay is minimized by 20% of TRSPC, 6.66% of RPRS-EH, 66.6% of Multi-relay system model, 24% of EHA-CRD and 2.66% of HFDCSA-AMLP respectively in Fig. 9b for 3rd sub-channel. It is similar to the optimization algorithm-based analysis also. Moreover, while considering other performance metrics also, it has been portrayed that the suggested approach attained lower execution time and higher throughput than the conventional algorithms and techniques. When considering the without relay-based analysis, the suggested mechanism’s network capacity is enhanced by 4.76% of SCO, 7.14% of FDA, 5% of MRFO, and 7.38% of CBOA respectively in Fig. 8f for the 5th sub-channel. This is similar to the method-based analysis also. Thus, it has been confirmed that the recommended mechanism showed higher optimization capabilities at node 250, increasing energy efficiency by 40% than SCO and 27.27% than FDA even if there is no relay. Also, the other performance metrics also elucidated that the presented work provided better outcomes than the conventional algorithms and techniques.

### Performance estimation of the suggested prediction approach

**Fig. 10:**
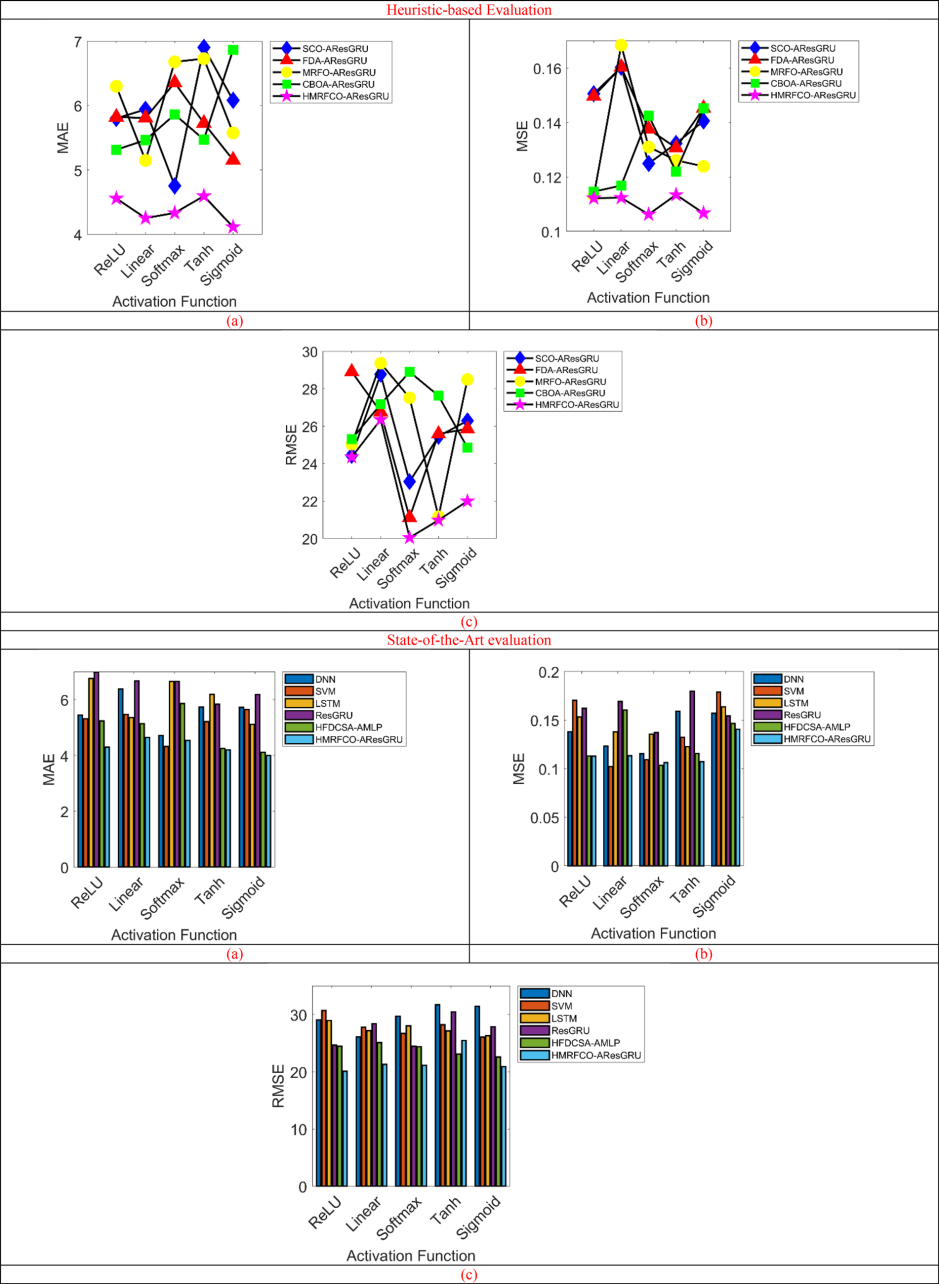
Performance estimation of recommended prediction mechanism over conventional heuristic algorithms and state-of-the-art techniques concerning “(**a**) MAE, (**b**) MSE, and (**c**) RMSE”.

The suggested prediction technique’s performance estimation is conducted in Fig. 10 over traditional algorithms and techniques by employing the activation function. Some of the effective activation functions such as “ReLU, linear, softmax, Tanh, and sigmoid” are considered in this analysis. Moreover, error measures such as MAE, MSE, and RMSE are supported for validating the recommended HMRFCO-AResGRU approach. This error-based analysis shows that the suggested HMRFCO-AResGRU mechanism attained very low error rates than the conventional techniques. In Fig. 10a, the HMRFCO-AResGRU model has lowest prediction error and it achieves 43% lower MAE compared to DNN. Figure 10b, shows the MSE. When taking the linear activation function in Fig. 10c, the RMSE of the presented prediction task is reduced by 9.54% of SCO-AResGRU, 1.14% of FDA-AResGRU, 13.35% of MRFO-AResGRU, and 3.05% of CBOA-AResGRU respectively. Thus, it has been confirmed that the presented higher prediction accuracy was demonstrated by the AResGRU based prediction model, which reduced RMSE by 24.96% when compared to ResGRU and by 33.5% when compared to LSTM. Moreover, the analysis shows that the suggested prediction mechanism has shown improved performance rates than the conventional prediction techniques. It has been proved that the offered work can support the D2D communication system without any errors.

### Overall performance estimation of the suggested prediction approach

Overall performance validation is performed for the suggested prediction technique over traditional algorithms and techniques. This analysis is shown in Table 6. By employing the error measures, this validation is conducted and the estimation showed that the recommended prediction mechanism attained very low error values than the conventional approaches. The suggested prediction technique’s MAE is relatively minimized by 43% of DNN, 41% of SVM, 27.75% of LSTM and 54.25% of ResGRU and 2.75% of HFDCSA-AMLP correspondingly. Thus, it has been ensured that the presented prediction mechanism is highly supportive of the D2D communication system than the other conventional approaches.

**Table 6:** Overall performance estimation of suggested prediction mechanism over traditional algorithms and techniques.

Terms	SCO-AResGRU^45^	FDA-AResGRU^46^	MRFO-AResGRU^47^	CBOA-AResGRU^50^	HMRFCO-AResGRU
Heuristic algorithm-based evaluation					
MAE	6.0814	5.1554	5.5754	6.8636	4.114
MSE	0.14055	0.14528	0.12388	0.14521	0.10672
RMSE	26.29		28.494	24.853	21.994

Terms	DNN^48^	SVM^49^	LSTM^30^	ResGRU^44^	HMRFCO-AResGRU
State-of-the-art-based evaluation					
MAE	5.7253	5.6407	5.1116	6.1788	4.0002
MSE	0.15706	0.17888	0.16365	0.1544	0.14055
RMSE	31.4	26.045	26.29	27.859	20.882

### Time complexity analysis of recommended system over conventional algorithms

The suggested scheme’s time complexity is evaluated over conventional algorithms and presented in Table 7. From nodes 50–250 this estimation is conducted for verifying the time complexity of the suggested system. This analysis clearly shows that the recommended scheme utilizes much less amount of time for computation than the other techniques. This increases the efficacy of the suggested framework. Moreover, it has been proved that the presented work overcame the conventional techniques in terms of time and increased the reliability of the system. The peak memory consumption during training of the AResGRU model is approximately 240 MB. The performance tested up to 250 nodes and 5 subchannels (Figs. 6 and 9), for ultra dense 5G/6G networks, we can extend up to 1000+ nodes also.

**Table 7:** Time complexity analysis of recommended system over traditional algorithms.

Terms	Time complexity (sec)
“Node-50”	
SCO^17^	0.1274
FDA^47^	0.0986
MRFO^14^	0.1002
CBOA^15^	0.0911
HFDCSA^35^	0.0923
Recommended HMRFCO	0.0878
“Node-100”	
SCO^17^	0.2163
FDA^47^	0.2244
MRFO^14^	0.2097
CBOA^15^	0.1989
HFDCSA^35^	0.1952
Recommended HMRFCO	0.1865
“Node-150”	
SCO^17^	0.2897
FDA^47^	0.3183
MRFO^14^	0.3003
CBOA^15^	0.2798
HFDCSA^35^	0.2743
Recommended HMRFCO	0.1865
“Node-200”	
SCO^17^	0.3976
FDA^47^	0.3462
MRFO^14^	0.3542
CBOA^15^	0.3442
HFDCSA^35^	0.3364
Recommended HMRFCO	0.3109
“Node-250”	
SCO^17^	0.4082
FDA^47^	0.4322
MRFO^14^	0.4211
CBOA^15^	0.3811
HFDCSA^35^	0.4021
Recommended HMRFCO	0.3672

Algorithm complexity:

HMRFCO: O (Population × Iteration × Dimensions).

AResGRU: O (Layers × neurons).

## Conclusion

A robust and effective system has implemented in this paper for performing the optimal relay selection and resource allocation in the D2D communication device by utilizing the hybrid heuristic approach-based deep learning. This work concentrated to enhance the efficiency and sum rate of the entire D2D system and cellular links. For this objective, a new HMRFCO was developed, where the conventional CBOA and MRFO techniques were integrated. Moreover, the suggested HMRFCO was employed for optimizing the multi-objective functions such as throughput, delay and network capacity, EE, and spectral efficiency to attain better network functionality. In this task, the dataset was created from numerous scenarios that were given to the AResGRU technique for predicting the optimal relay selection and resource allocation techniques. Here, the parameters in the AResGRU approach were optimally determined by the same HMRFCO approach. The final prediction of the optimal resource allocation and relay selection was obtained by the AResGRU approach. The experiments were carried out to validate the functionality rate of the presented system. The recommended joint optimal relay selection and resource allocation approach’s success rate was highly enhanced by 4.12% of TRSPC, 9.27% of RPRS-EH, 3.09% of Multi-relay system model, 7.21% of EHA-CRD, and 2.06% of HFDCSA-AMLP correspondingly when considering the number of sub-channels is 5 in the relay-based analysis. Thus, it has been confirmed that the presented joint optimal resource allocation and relay selection scheme attained 9.27% improvement in success rate and 6.66% reduction in delay than the conventional mechanisms such as RPRS-EH and the Multi-relay system. The presented work applies to numerous real-time transmission devices including 5G communication systems, multimedia and content sharing, E-health, gaming, local social networking, etc. Though the presented work provides very promising solutions, the prediction network may encounter poor generalization capabilities. Hence, in future work, a very effective hybrid or ensemble-based deep learning approach will be introduced to provide more generalization. UAV assisted D2D Communication can extend the coverage area, increase the Energy Efficiency, and throughput. Integrate D2D communication with RIS (Reconfigurable Intelligent Surface) to increase the EE and link reliability, and Multi-access Edge Computing (MEC) to reduce the load on central base station and low latency. The Reinforcement learning-based policy tuning to enable real-time adaptation of these parameters for even greater flexibility and responsiveness.

## Data Availability

The datasets used and/or analysed during the current study are available from the corresponding author on reasonable request.
